# Preventive Effects of Cinnamon Leaf Nanosuspension and Byproducts on Type II Diabetes and Parkinson’s Disease in Rat Models

**DOI:** 10.3390/antiox15020195

**Published:** 2026-02-02

**Authors:** Jin-Wei Lee, Chen-Te Jen, Baskaran Stephen Inbaraj, Bing-Huei Chen

**Affiliations:** Department of Food Science, Fu Jen Catholic University, New Taipei City 242062, Taiwan; fzmango77@gmail.com (J.-W.L.); d11641002@ntu.edu.tw (C.-T.J.); 138547@mail.fju.edu.tw (B.S.I.)

**Keywords:** cinnamon leaf nanosuspension, hydrosol, type II diabetes, Parkinson’s disease, preventive effect, rat model

## Abstract

Cinnamon leaves, an important source of cinnamaldehyde, have been shown to possess various pharmacological functions. A liposome-derived nanosuspension (CN), hydrosol (CH) and powder in water (CP) prepared from cinnamon leaves were explored for their preventive effects on type II diabetes (T2D) and Parkinson’s disease (PD) in rat models. Fifteen compounds were determined by UPLC-MS/MS, with cinnamaldehyde being predominant. CN with a mean particle size at 22.6 nm was prepared by mixing extract, lecithin, Tween 80, soybean oil and water with an optimal ratio, while hydrosol was prepared by steam distillation. A high storage and gastrointestinal stability were observed for CN. Rats were pre-fed with CN, CH and CP separately for 4 weeks, followed by induction with T2D or PD, with all three groups showing better preventive effects on T2D given the number of injections for T2D induction. Additionally, the OGTT, HOMA-IR, TXB2 and aPTT levels were significantly higher in the induction group than in the CN, CH and CP groups, revealing an effective prevention of T2D and cardiovascular disease. For PD prevention, CN was the most effective in improving muscle stiffness based on the catalepsy test and elevation of dopamine, tyrosine hydroxylase, serotonin, mitochondrial DNA and antioxidant enzymes, accompanied by a decline in α-synuclein and malondialdehyde.

## 1. Introduction

*Cinnamomum osmophloeum* (*C. osmophloeum*), a traditional and endemic plant grown in Taiwan, belongs to the *Cinnamomum* genus and Lauraceae family. Because of the presence of abundant bioactive compounds and the unique aroma in *C. osmophloeum* leaves (cinnamon leaves), they are often used as raw materials for the production of functional foods and spices [1]. Published reports have shown that cinnamon leaves from Taiwan contain various bioactive compounds, with cinnamaldehyde (CA) showing the highest content, followed by *trans*-cinnamic acid, eugenol, cinnamyl alcohol and benzoic acid [2,3]. Most importantly, the Taiwan cinnamon leaf extracts have been demonstrated to be effective in improving chronic diseases such as type II diabetes (T2D) and Parkinson’s disease (PD) in rat models [2,3], which can be attributed to the presence of bioactive compounds, especially the dominant CA.

Diabetes, an abnormal chronic disease, can be divided into types I and II, with the former mainly resulting from the impairment of β cells in pancreatic islets for subsequent inhibition of insulin secretion, and the latter caused by insulin resistance for subsequent high-level insulin production and then low-level insulin secretion, leading to hyperglycemia [2]. It is well known that the main function of β cells in pancreatic islets is pivotal to secrete insulin for glucose uptake by the muscle, adipose and liver cells for energy production [4]. Long-term hyperglycemia has been reported to be closely associated with the occurrence of chronic diseases such as kidney, cardiovascular and neurological diseases [5]. Currently, T2D accounts for 90–95% of diabetic patients worldwide [6]. Based on a report issued by the International Diabetes Federation [7], the number of diabetic patients has exceeded 500 million worldwide and may reach 643 million by 2030. Currently, the first-line drug used for the treatment of T2D is metformin [8]. However, many side effects, such as gastrointestinal disturbance, loss of appetite, anemia, liver inflammation and skin rash, may occur depending on the individual response to metformin [9]. Thus, it is pivotal to develop a plant-derived product to treat patients with T2D without side effects [10].

Another important age-related neurodegenerative disease, PD, is becoming more prevalent worldwide due to the sharp increase of the aging population in recent years [11]. According to a report issued by the World Health Organization [12], the number of patients with PD will rise to 18 million in 2030. PD, a degenerative disease caused by an inadequate production of dopamine because of a malfunction of the basal ganglia and substantia nigra in the brain, as well as a subsequent buildup of α-synuclein in neurons, leading to Lewy body formation, is a movement disorder [13]. Many factors, such as the overproduction of free radicals, the elevation of oxidative stress and the presence of high levels of iron and low levels of antioxidants, have been reported to play a significant role in the occurrence and progress of PD [14]. Levodopa, a widely used drug for the treatment of PD patients, can increase dopamine levels in the brain [15]. However, many side effects, such as vomiting, appetite loss, nausea, low blood pressure and confusion, may occur [15,16]. Consequently, like T2D, the development of a plant-derived product for the treatment of PD patients without side effects is imperative [16].

To enhance the treatment efficiency of both T2D and PD in vivo, the employment of an encapsulation technique to elevate the stability of bioactive compounds under gastrointestinal condition is extremely important [17,18,19]. Among the various encapsulation techniques, the development of an appropriate nanosystem such as nanoliposome or nanoemulsion has been widely applied to the treatment of chronic diseases [20,21]. Nanoliposomes are lipid-soluble spherical vesicles with particle sizes in the range of 10–200 nm, which are formed by the self-assembly of amphipathic molecules like phospholipids, consisting of a hydrophilic head and two hydrocarbon chains to form a membrane [21]. This phospholipid membrane is arranged with hydrophilic head groups facing the outer aqueous environment and the hydrocarbon tails facing the hydrophobic interior. Thus, nanoliposomes are capable of encapsulating hydrophobic compounds between lipid bilayers, as well as hydrophilic compounds in the aqueous core [21]. More importantly, nanoliposomes or nanoemulsions can be designed to be more selective and specific for drug delivery to the brain through binding with receptors at the blood–brain barrier, there by enhancing the treatment efficiency of neurodegenerative diseases [22,23].

In a previous study dealing with cinnamon leaves, Huang and Chen [2] developed a cinnamon leaf nanoemulsion composed of cinnamon leaf extract, soybean oil, Tween 80, lecithin and deionized water, with a mean particle size, polydispersity index, zeta potential and encapsulation efficiency of 36.6 nm, 0.222, −42.6 mV and 91.2%, respectively. Furthermore, it was found effective in reducing the levels of fasting blood glucose (FBG), oral glucose tolerance test (OGTT) value, serum insulin and insulin resistance index in rats. In another study, Wang et al. [3] developed a similar cinnamon leaf nanoemulsion, which was demonstrated to improve PD in rats through an elevation of the levels of dopamine, tyrosine hydroxylase and antioxidant enzymes, including superoxide dismutase (SOD), catalase (CAT) and glutathione peroxidase (GSH-Px), as well as a decrease of α-synuclein and malondialdehyde (MDA) generated from lipid peroxidation and degradation. More recently, a cinnamon leaf nanoemulsion with a mean particle size of 17.1 nm, polydispersity index of 0.236, zeta potential of −42.7 mV and encapsulation efficiency of 90.8% was prepared and demonstrated to improve short-term and long-term memory in rats with a dose-dependent amelioration of symptoms associated with Alzheimer’s disease (AD) [23]. It was also shown to reduce amyloid-β 40, BACE1 and 8-oxodG in the hippocampus, and AchE and MDA in rat cortices with a concomitant increase in antioxidant enzymes (SOD, CAT, GSH-Px) in rat cortices and livers, as well as dopamine in rat striatum. More importantly, the incidence of AD in rats leading to the development of PD was also verified in the same study [23]. Collectively, the typical AD biomarkers tau and Aβ were present in patients with PD, while α-synuclein, the main component of Lewy bodies in PD patients, was also found in AD patients [24]. Some common pathological mechanisms involved in AD and PD include mitochondrial dysfunction, neuroinflammation, synapse dysfunction, oxidative stress, autophagy dysfunction, calcium ion imbalance and the formation of advanced glycation end products [23,24,25].

A close association has been shown not only between AD and PD, but also between T2D and PD, as well as between T2D and AD, as T2D patients with insulin resistance were shown to have a symptom of long-term hyperglycemia, which is the principal factor in the development of neurodegenerative diseases such as AD and PD [23]. For instance, a decrease in dopamine levels and a rise in mitochondrial dysfunction were found in diabetic mice, with the latter elevating α-synuclein levels in Lewy bodies [26,27], while the highly prevalent advanced glycation end products in hyperglycemia patients coexisted with α-synuclein in the Lewy body [28]. In addition, the diabetic peripheral neuropathy that affects the motor functions in diabetic patients are more prevalent in PD patients [29]. Thus, a drug capable of alleviating T2D should improve PD. However, the presence of the blood–brain barrier (BBB), a nature brain barrier which can prevent harmful substances in the blood from entering brain, may prevent bioactive compounds from entrance. Therefore, by selecting lipophilic bioactive compounds with molecular weight (MW) < 400 and encapsulating into a liposome-derived nanosuspension, it is possible to cross the BBB and thereby improve neurogenerative diseases, as pointed out by Chen et al. [23]. This prompted us to verify the application of a cinnamon leaf nanosystem and the byproducts CH and CP for the prevention of T2D and PD in rat models.

Furthermore, owing to the rising aged population, “prevention is better than cure” is always invaluable advice which necessitates the development of strategies for the prevention of chronic diseases. Consequently, in this study, the CN and its byproducts, CH and CP, were prepared from cinnamon leaves and pre-fed separately to rats for 4 weeks prior to T2D or PD induction, followed by continued feeding for another 5 weeks for evaluation of their preventive effects on T2D and PD. It is worth pointing out that this is the first study to explore the preventive effects of CN, CH and CP on T2D and PD in rat models for a possible treatment of T2D and PD simultaneously.

In this study, we measured the levels of FBG, OGTT and insulin and performed a homeostatic model assessment of insulin resistance (HOMA-IR) in streptozotocin (STZ)-induced diabetic rats, followed by the measurement of several biochemical parameters, including triglyceride (TG) and total cholesterol (TC) for the evaluation of cardiovascular function, aspartate aminotransferase (AST) and alanine aminotransferase (ALT) for liver function, uric acid (URIC), and blood urea nitrogen (BUN) and creatinine (CREA) for kidney function. Furthermore, thromboxane B2 (TXB2) and prothrombin (PT), a blood test measuring how long it takes for blood to clot, and activated partial thromboplastin time (aPTT), measuring the time for blood to clot in the presence of a reagent, were measured in T2D rats. For PD rats, the levels of dopamine, serotonin, tyrosine hydroxylase and α-synuclein, as well as the mitochondria deoxyribonucleic acid (mtDNA) copy number, were determined. In addition, the activities of antioxidant enzymes, including SOD, CAT and GSH-Px, as well as the lipid oxidation product MDA, were measured. Finally, the catalepsy test was performed for the evaluation of muscle stiffness in PD rats. The success of this study can lay a foundation of preventive medicine through the intake of cinnamon leaf nanosuspension and byproducts prior to the occurrence of chronic diseases such as T2D and PD.

## 2. Materials and Methods

### 2.1. Materials

The cinnamon leaves were harvested in Taitung County, Taiwan, in November 2023 and transported to our lab. Of the 15 standards, eugenol, kaempferol, quercetin, kaempferol 3-β-D-glucopyranoside, quercetin-3-O-glucoside, rutin and p-coumaric acid were purchased from Sigma-Aldrich (St. Louis, MO, USA), while trans-cinnamic acid, cinnamaldehyde, cinnamyl alcohol, benzoic acid and caffeic acid were from Chem Service Inc. (West Chester, PA, USA). Quercetin-3-O-galactoside and 5-O-caffeoylquinic acid were from Biopurity Phytochemicals Ltd. (Chengdu, China), and coumarin was from Accustandard (St. New Haven, CT, USA).

Solvents such as 99% ethanol and n-hexane were obtained from Sigma-Aldrich, while methanol from Merck (Darmstadt, Germany). Glacial acetic acid (≥99.7%) was from Thermo Fisher Scientific Inc. (San Jose, CA, USA). Deionized water was collected from a Milli-Q water purification system (Millipore Corporation, Bedford, MA, USA). Soybean oil was procured from Taiwan Sugar Co. (Tainan City, Taiwan), while Tween 80 and lecithin were, respectively, obtained from Yuba Enterprise Co., Ltd. (Taipei, Taiwan) and Jen Fang Co. (Taipei, Taiwan). Potassium dihydrogen phosphate and phosphotungstic acid were also purchased from Sigma-Aldrich, while the carbon-coated copper grid was from Electron Microscopy Sciences (Hatfield, PA, USA).

The ACQUITY UPLC system coupled with a triple quadrupole tandem mass spectrometer was from Waters (Milford, MA, USA), while Luna Omega C18 column (100 × 2.1 mm ID, particle size 1.6 μm) was from Phenomenex (Torrance, CA, USA) and the N1200A model rotary evaporator from Eyela Co. (Tokyo, Japan). The DC400H model ultrasonicator was from Hua-Hsiah Scientific Co. (Taipei, Taiwan), while 5810R model normal centrifuge and Heraeus Fesco21 model microcentrifuge machines were, respectively, from Eppendorf Co. (Hauppauge, NY, USA) and Thermo Fisher Scientific Co. The 90 Plus model dynamic light scattering particle size analyzer was from Brookhaven Instruments (Holtsville, NY, USA), while the SZ-100 model zeta potential analyzer was from Horiba Scientific Co. (model SZ-100, Kyoto, Japan).

For animal experiments, STZ, nicotinamide (NA) and dimethyl sulfoxide (DMSO) were purchased from Sigma-Aldrich, while sunflower oil was from Standard Foods Co. (Taoyuan, Taiwan). Serum insulin and thromboxane (TXB2), as well as contents of dopamine, α-synuclein, tyrosine hydroxylase and serotonin, were measured by commercial kits from Fine Biotech Co. (Wuhan, China), while mitochondrial deoxyribonucleic acid (mtDNA) copy number was quantitated using the Absolute Rat Mitochondrial DNA Copy Number Quantification (ARMQ) qPCR assay kit from ScienCell Research Laboratories (Carlsbad, CA, USA). Activities of antioxidant enzymes, including SOD, CAT and GSH-Px, as well as the lipid oxidation product MDA, were measured using commercial kits from Cayman Chemical (Ann Arbor, MI, USA).

### 2.2. Processing of Cinnamon Leaves and Hydrosol

One kg of cinnamon leaves was washed and dried in an oven at 60 °C for 2 h. Then, the dried cinnamon leaves were ground into powder (545 g) for subsequent experiments, with the moisture contents in fresh and oven-dried leaves being 46.5% and 5.4%, respectively. Additionally, a steam distillation method was used to obtain CH (30 L) by collecting 8 kg of cinnamon leaves harvested in Taitung County, Taiwan and mixing with 50 L of deionized water for distillation at 100 °C for 3 h.

### 2.3. Determination of Bioactive Compounds in Cinnamon Leaves and Hydrosol by UPLC-MS/MS

The bioactive compounds in dried cinnamon leaves were extracted and analyzed by UPLC-MS/MS using a method by Wang et al. [3] with a slight modification. Briefly, one gram of cinnamon leaf powder was mixed with 30 mL of 80% ethanol, after which this mixture was subjected to sonication for 2 h at 60 °C and then centrifugation for 20 min at 4000 rpm (25 °C). Following repeating this step 3 times, the supernatants were pooled, followed by filtration through a filter paper, solvent evaporation with nitrogen gas, dissolving the residue in 80% ethanol (10 mL) and filtering through a 0.22 μm membrane filter for UPLC-MS/MS analysis. But for CH containing about 97% water, it was directly filtered through a 0.22 μm membrane filter for UPLC-MS/MS analysis.

A mobile phase of 0.025% acetic acid in water (A) and 0.025% acetic acid in methanol (B) with a gradient mode was used: 83% A and 17% B in the beginning, changed to 80% A and 20% B in 1 min, 60% A and 40% B in 5 min, 45% A and 55% B in 10 min, 1% A and 99% B. in 14 min, and then returned to the original ratio. Using a Luna Omega C18 column (100 × 2.1 mm ID, particle size 1.6 μm) and column temperature at 30 °C with flow rate at 0.3 mL/min, 15 compounds, including eugenol, *trans*-cinnamic acid, kaempferol, cinnamaldehyde, cinnamyl alcohol, quercetin, kaempferol 3-β-D-glucopyranoside, quercetin-3-O-glucoside, quercetin-3-*O*-galactoside, rutin, benzoic acid, caffeic acid, coumarin, *p*-coumaric acid and 5-*O*-caffeoylquinic acid, were separated within 14 min and detected using a multiple reaction monitoring (MRM) mode with a negative ion mode as described by Huang and Chen [2]. However, both peaks of quercetin-3-*O*-glucoside and quercetin-3-*O*-galactoside were overlapped. Moreover, the method validation for this UPLC-MS/MS method was previously reported [2], with the repeatability (intra-day variability), intermediate precision (inter-day variability) and recovery being determined following the Taiwan Food and Drug Administration (TFDA) recommendations [30], while both LOD and LOQ were measured following the International Conference on Harmonization (ICH) guidelines [31]. For quantitation, all 15 identified compound standards with 8 concentrations (10, 20, 50, 100, 200, 300, 400, 500 ng/mL) of each standard were prepared and subjected to UPLC-MS/MS analysis as shown above; then, the calibration curves of each standard were obtained by plotting peak area against concentration. Next, the linear regression equation of each standard was used to quantify the amount of each compound [3].

### 2.4. Preparation of Liposome-Derived Cinnamon Leaf Nanosuspension (CN)

The preparation of CN with specific components and composition was based on a method reported by Wang et al. [3]. As CA is the dominant compound in cinnamon leaf extract, the CN containing CA at 10,000 µg/mL was thus prepared by mixing 0.6 g of Tween 80 (6%), 0.2 g of lecithin (2%) and 0.1 g of soybean oil (1%) in a flask, followed by dissolving in 99% ethanol, homogenizing thoroughly and adding 20 mL of cinnamon leaf extract. Next, this mixture was evaporated to form a thin film in the flask bottom under reduced pressure, after which deionized water (9.1 mL) was added for subsequent sonication for 30 min to obtain a dark green-colored CN.

### 2.5. Determination of CN Properties

Both polydispersity index and mean particle size of CN were measured using a dynamic light scattering (DLS) instrument by collecting a portion (100 μL), followed by 10-fold dilution with potassium dihydrogen phosphate buffer solution (25 mM, pH 5.5) and pouring into a colorimetric tube for measurement. The zeta potential was determined by collecting a portion (100 μL), diluting 10 times with deionized water and pouring into a folded capillary tube for measurement at 25 °C.

Additionally, a transmission electron microscope (TEM) was used to measure shape and nanoparticle size of CN by diluting with deionized water, followed by collecting a portion (100 μL), dropping on a copper grid (carbon-coated) for 90 sec, removing the excessive sample with a filter paper, negative staining with phosphotungstic acid (20 μL), removing the excessive stain and then drying in an oven overnight for imaging.

For the determination of encapsulation efficiency, a portion of CN (100 μL) was collected and mixed with n-hexane (400 μL), after which this mixture was shaken so that CA dissolved into the n-hexane phase in the upper layer. Likewise, a portion of CN (100 μL) was collected and mixed with 99% ethanol (400 μL) thoroughly for subsequent ultrasonication for 2 h to dissolve total CA. Then, both free CA and total CA were quantified using the same UPLC column and separation condition as described above, but with detection at 280 nm. The encapsulation efficiency was then measured using a formula as described by Wang et al. [3].

### 2.6. Determination of Storage Stability and Stability in Simulated Gastric and Intestinal Fluid

For the storage stability study, the CN was stored for 12 weeks at 4 °C, during which a portion was collected every week for the measurement of zeta potential, mean particle size and polydispersity index following the same methods as shown above.

The stability of CN in simulated gastric and intestinal fluid was determined based on methods reported by Hsu and Chen [32] and Ye et al. [33]. Initially, gastric fluid was prepared by mixing 2 g/L sodium chloride and 3.2 g/L pepsin in deionized water with a subsequent adjustment of pH to 1.5 using 1 M hydrochloride acid, while the intestinal fluid was prepared by mixing 8.09 g/L monopotassium phosphate and 5.16 g/L bile salts in deionized water with a subsequent adjustment of pH to 7.5 using 0.1 M sodium hydroxide.

For the determination of CN stability under gastric conditions, 4 mL of CN was mixed with 16 mL of simulated gastric fluid in a beaker, followed by stirring at 100 rpm for 2 h at 37 °C, collecting 1 mL from the mixture every 30 min and pouring into a centrifuge tube. After centrifuging at 1000 rpm (4 °C), the supernatant was collected and diluted 50-fold with deionized water for zeta potential measurement as well as diluted 10-fold separately with 25 mM potassium dihydrogen phosphate buffer for particle size measurement.

For the determination of CN stability under intestinal conditions, 5 mL of gastric-digested CN sample was mixed with 5 mL of simulated intestinal fluid in a beaker, followed by stirring at 100 rpm at 37 °C for 2 h, collecting 1 mL from the mixture every 30 min and centrifuging at 1000 rpm (4 °C). Then, the supernatant was collected and diluted 10-fold with deionized water for zeta potential measurement as well as diluted 5-fold separately with 25 mM potassium dihydrogen phosphate buffer for particle size measurement. Additionally, the encapsulation efficiency was determined by separately collecting 100 µL of supernatant after 2 h incubation with gastric fluid and intestinal fluid and following the same procedure as described in Section 2.5.

### 2.7. In Vitro Release of CA Under Gastrointestinal Condition

The in vitro release of CA under gastrointestinal conditions was determined based on a report by Hsu and Chen [32], using the same gastric fluid and intestinal fluid as described in Section 2.6. The in vitro release under gastric conditions was determined by mixing 2 mL of CN with 8 mL of gastric fluid in a beaker and stirring at 100 rpm for 4 h at 37 °C. Then, 0.5 mL was collected every 30 min, centrifuged at 1000 rpm (4 °C); the supernatant collected and diluted 100-fold with methanol for subsequent CA analysis by UPLC. For in vitro release under intestinal conditions, 2 mL of CN was mixed with 8 mL of intestinal fluid, followed by stirring at 100 rpm for 4 h at 37 °C, collecting 0.5 mL every 30 min, centrifuging at 1000 rpm (4 °C), collecting the supernatant and diluting 100-fold with methanol for subsequent CA analysis by UPLC.

### 2.8. Animal Experiment

Initially, the Institutional Animal Care and Use Committee (IACUC) of Fu Jen University (New Taipei City, Taiwan) approved all the animal experiments, which followed the standard experimental animal operation procedures. Overall, 30 male Wistar rats (6-week-old, 250 g) for the T2D study and 40 male Sprague-Dawley rats (6-week-old, 250 g) for the PDstudy were purchased from BioLASCO Co. Ltd. (Taipei, Taiwan). After transportation to the Fu Jen University Animal Center, all these rats were housed in individual ventilation cages with the relative humidity at 55 ± 10%, the temperature at 21 ± 2 °C and the day/light cycle for 12 h. Prior to the animal experiment, a permission document (approval number A11318) for performing T2D and PD issued by Fu Jen University Animal Care and Use Committee was obtained, with the experimental steps following approved guidelines. All rats were fed with standard rodent chow diet (LabDiet Co., St Louis, MO, USA) and water ad libitum during the experiment, with the water, food intake and body weight being recorded over a period of 9 weeks. Also, environmental conditions including lighting, humidity, temperature, noise and air quality levels were standardized in all cages during the entire experiment, and environmental enrichment in the form of wooden sticks and bedding materials was provided to have natural behavior and reduce stress.

One rat was regarded as one experimental unit, and the rats were randomly placed in individual standard polycarbonate cages with stainless steel wire lids (one rat per cage) by the animal center personnel for subsequent numbering with control group first, followed by the induction group and treatment groups. Both the position and numbering order of cages were maintained in an open rack, and all researchers involved in this study were informed of group allocation and order of cages during different stages of experiment including allocation, conduct of experiment, outcome assessment and data analyses to minimize potential confounders on experimental outcome. The overall health of rats was monitored once in two days by the animal center personnel.

To reduce handling stress, rats were gently grasped by the tail and supported with the opposite hand while removing from the cage and subsequently calmed by gentle stroking before feeding. In addition, the apparent signs of pain in rats, including vocalization, aggressive or defensive behavior, social withdrawal or self-isolation, were effectively managed with subcutaneous injection of ketoprofen (2–3 mg/kg). It was also established to euthanize rats by CO_2_ inhalation if the humane endpoints were encountered including rapid weight loss, severe weakness with an inability to eat/drink, paralysis and visible damage to vital organs. However, no expected or unexpected adverse events occurred during the study.

#### 2.8.1. Type II Diabetes

**Figure 1:**
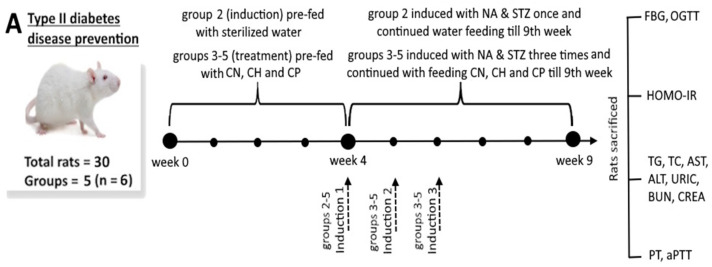

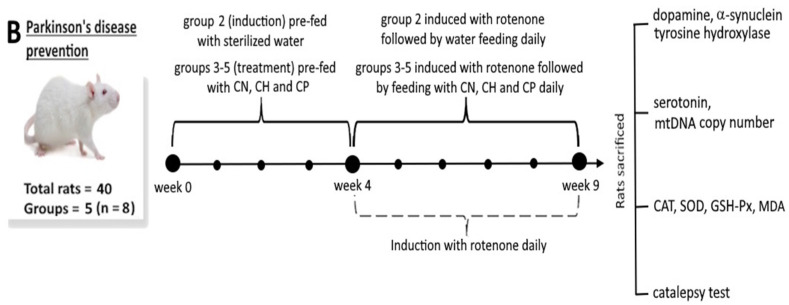
A schematic diagram illustrating the study design of animal experiments for prevention of T2D (**A**) and PD (**B**) in rat models. T2D, type II diabetes; PD, Parkinson’s disease; CN, cinnamon leaf nanosuspension; CH, hydrosol prepared by steam distillation of cinnamon leaves; CP; cinnamon leaf powder dispersed in water; NA, nicotinamide; STZ, streptozotocin; FBG, fasting blood glucose; OGTT, oral glucose tolerance test; HOMA-IR, homeostatic model assessment of insulin resistance; TG, triglycerides; TC, total cholesterol; AST, aspartate aminotransferase; ALT, alanine aminotransferase; URIC, uric acid; BUN, blood urea nitrogen; CREA, creatinine; PT, prothrombin time; aPTT, activated partial thromboplastin time; mtDNA, mitochondrial deoxyribonucleic acid; CAT, catalase; SOD, superoxide dismutase; GSH-Px, glutathione peroxidase; MDA, malondialdehyde.

A method based on Huang and Chen [2] was used to induce T2D in rats. Both STZ and NA used for induction were separately dissolved in physiological saline containing sodium citrate buffer at 0.1 M (pH 4.5). Figure 1A shows the schematic diagram illustrating the animal experiment design for prevention of T2D in a rat model. Wistar rats were used for T2D study as their metabolism can be manipulated to develop human T2D symptoms like hyperglycemia and insulin resistance through feeding with high-fat diets and injecting with STZ. Rats (7 weeks old) were fed with samples including CN, CH and CP separately for 4 weeks. Then, rats were fasted for 12 h before measuring FBG in serum, followed by hyperglycemia induction starting at the first day of the 5th week and measuring FBG at the first day of the 6th week. The induction was conducted by injecting NA intraperitoneally at a dose of 230 mg/kg body weight (bw), followed by injecting STZ at 65 mg/kg bw 15 min later. Then, a high-fat diet containing 60% fat was started to feed rats for 5 weeks. When the FBG level > 200 mg/dL, this implied a successful induction of T2D. Five groups with 6 rats each were used, and as no rats died, 6 rats per group (*n* = 6) was used for data analysis: (1) normal control (NC): rats were fed with deionized water by tube feeding each day for 9 weeks; (2) induction (IN): rats fed with deionized water by tube feeding each day for 4 weeks, followed by the induction of T2D once at the first day of the 5th week and continuing feeding until the 9th week; (3) cinnamon leaf nanosuspension (CN): rats fed with CN containing CA at 60 mg/kg bw each day for 4 weeks, followed by the induction of T2D at the first day of the 5th, 6th and 7th week for 3 times, and continuing feeding until the 9th week, (4) cinnamon hydrosol (CH): rats fed with CH at 11.9 mg/kg bw each day for 4 weeks, followed by the induction of T2D at the first day of the 5th, 6th and 7th week for 3 times, and continuing feeding until the 9th week; (5) cinnamon powder (CP): rats fed with CP at a dose of 0.5 g/kg bw each day for 4 weeks, followed by the induction of T2D at the first day of the 5th, 6th and 7th week for 3 times, and continuing feeding until the 9th week. All the doses were selected based on a previous study by Huang and Chen [2] and the results of preliminary tests in our lab. The number of inductions was based on FBG level for comparison with the induction group, which was only induced once for successful induction of T2D.

The FBG level was measured once a week following 12 h fasting using a glucometer (GE100, New York, NY, USA) by collecting blood from the tail vein of rats, while the OGTT (oral glucose tolerance test) was carried out at the first day of the 9th week by feeding rats with samples of each treatment (groups 1–5) for 30 min, followed by tube feeding with glucose at a dose of 1 g/kg bw for the subsequent measurement of blood glucose level at 0, 30, 60 and 120 min. Meanwhile, the serum insulin was determined using an antibody radioimmunoassay kit, and the homeostatic model assessment of insulin resistance (HOMA-IR) was measured using a formula as described by Huang and Chen [2]. Additionally, the biochemical parameters TG, TC, AST, ALT, URIC, BUN and CREA were analyzed by a VetTest automated clinical chemistry analyzer (Westbrook, ME, USA), while TXB2 was measured by a commercial kit, while PT and aPTT were measured using plasma collected from blood with an automated benchtop analyzer (Stago Compact Max, France).

#### 2.8.2. Parkinson’s Disease

Figure 1B shows the schematic diagram illustrating the animal experiment design for prevention of PD in a rat model. Rats (7 weeks old) were initially fed with samples each day, including CN, CH and CP dispersed in water separately for 4 weeks. At the first day of the 5th week, rats were induced to generate PD by injecting rotenone (dissolved in 2% dimethyl sulfoxide and 98% sunflower oil) at 2 mg/kg bw subcutaneously, with rats being continued to be fed with samples and injected with rotenone each day for 5 weeks. Sprague Dawley rats were used for PD study as they can closely mirror human populations, contribute to reliable results and attain high performance in preclinical studies. Compared with prevention experiments for T2D (*n* = 6), a higher number of rats per group (*n* = 8) was chosen for prevention experiments for PD as some rats may die due to daily injections of rotenone from 5th week to 9th week (Figure 1). Overall, 40 rats were divided into 5 groups with 8 rats each, and as no rats died, 8 rats per group (*n* = 8) were used for data analysis. Group 1 (normal control group, NC): rats were fed with deionized water by tube feeding each day for 4 weeks, followed by injecting sunflower oil (98%) and dimethyl sulfoxide (2%) subcutaneously at 1 mL/kg bw and then tube feeding with deionized water at 10 mL/kg bw for 5 weeks; Group 2 (induction group, IN): rats were fed with deionized water by tube feeding each day for 4 weeks, followed by injecting rotenone (dissolved in sunflower oil) subcutaneously at 2 mg/kg bw each day and then tube feeding with deionized water at 10 mL/kg bw each day for 5 weeks; Group 3 (nanosuspension group, CN): rats were fed with CN by tube feeding 60 mg/kg bw each day for 4 weeks, followed by the induction of PD, and continuing feeding with CN each day for 5 weeks; Group 4 (hydrosol group, CH): rats were fed with CH by tube feeding each day at 11.9 mg/kg bw for 4 weeks, followed by the induction of PD, and continuing feeding with CH each day for 5 weeks; Group 5 (powder dispersed in water group, CP): rats were fed with CP by tube feeding at 0.5 g/kg BW each day for 4 weeks, followed by induction of PD and continuing feeding with CP each day for 5 weeks. All the doses were selected based on animal experiments in several published reports and the results of preliminary tests in our lab. After feeding for 9 weeks, rats were sacrificed, and the brain striatums were collected for determination of dopamine, α-synuclein and tyrosine hydroxylase contents [3]. Also, the serotonin content and mtDNA copy number were analyzed by commercial kits. mtDNA is a circular multi-copy genome located in the mitochondria playing a key role in cellular energy production. Early detection of changes in the number of mtDNA will prevent and delay the occurrence of aging-associated chronic diseases such as PD, as the ability of brain cells to produce energy depends on the integrity and copy number of mtDNA. Using the rat mtDNA copy number quantitative qPCR detection kit for comparison of the rat mtDNA copy number, the improvement of PD can be assessed. Thus, in our study, the absolute number of mtDNA in the cell nucleus was determined by ScienCell’s commercial kit to obtain the relative number of mtDNA in brain cells. Additionally, the antioxidant enzyme activities, including the CAT, SOD and GSH-Px, as well as the lipid oxidation product MDA in the midbrain of rats, were measured using commercial kits [3].

Although the doses of CN (60 mg/kg bw) and CH (11.9 mg/kg bw) were based on the CA content in cinnamon leaf extract and hydrosol (Table 1), the dose of CP (0.5 g/kg bw) was based on the amount of raw cinnamon powder (0.5 g) suspended in deionized water (10 mL) for studying the comparative preventive effects of CN, CH and CP on T2D and PD rats.

#### 2.8.3. Catalepsy Test

A bar test was used to perform the catalepsy test by placing the front paws of a rat on an elevated bar with the hind paws remaining on the floor and measuring time length of the rat to remove or lift one paw from the bar [3]. This test can be used to test motor coordination and muscle stiffness of rats and can be an index of the catalepsy intensity.

### 2.9. Statistical Analysis

All the analyses were done in triplicate, and the data were subjected to an analysis of variance (ANOVA) and Duncan’s multiple range test for significant difference by comparing the mean values at a probability value of *p* < 0.05 using a statistical analysis system (SAS) with a software package (version 6, Cary, NC, USA).

## 3. Results and Discussion

### 3.1. Determination of Cinnamaldehyde and the Other Compounds in Cinnamon Leaves and Hydrosol

**Figure 2:**
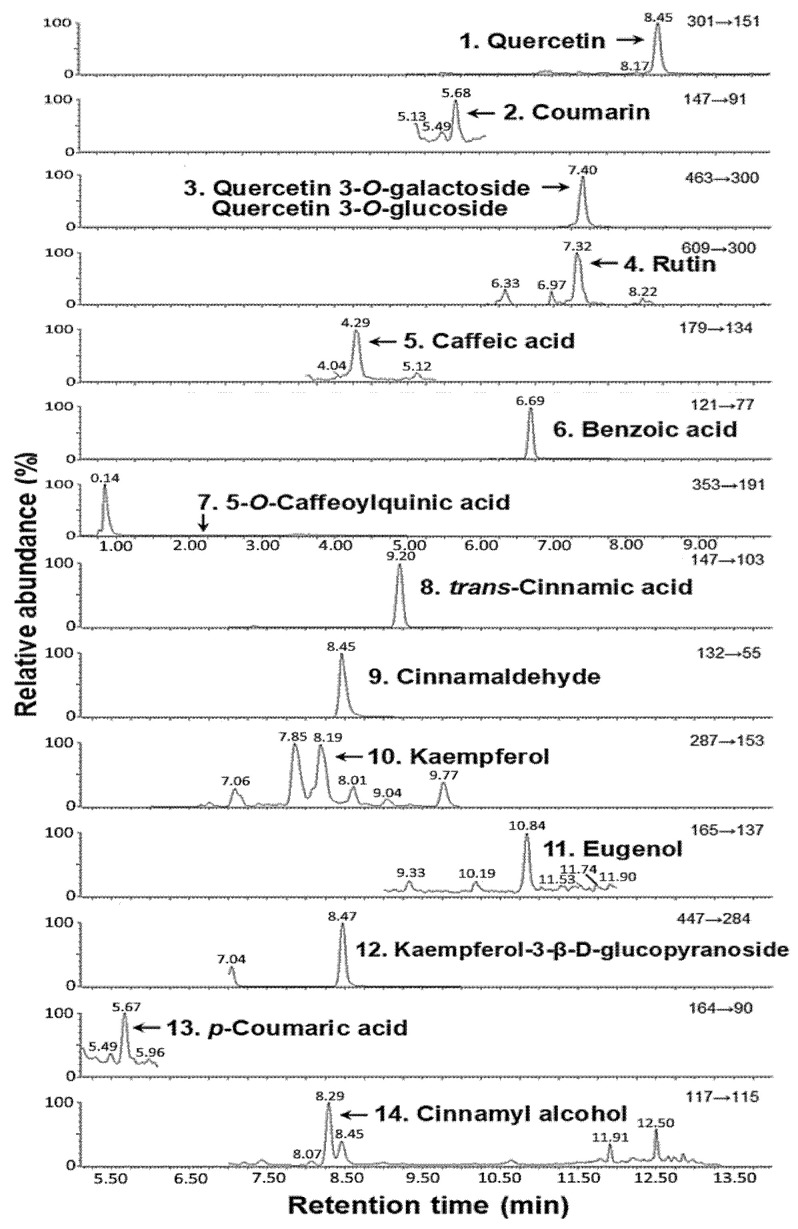
Ultra-performance liquid chromatography-mass spectrometry/mass spectrometry (UPLC-MS/MS) chromatogram of various bioactive compounds in cinnamon leaves detected by multiple reaction monitoring (MRM) mode. The UPLC-MS/MS method is described in Section 2.3.

Following the extraction, identification and quantitation procedures described in the method section, 15 compounds, including quercetin, coumarin, quercetin-3-O-glucoside, quercetin-3-O-galactoside, rutin, caffeic acid, benzoic acid, 5-O-caffeoylquinic acid, trans-cinnamic acid, cinnamaldehyde, kaempferol, eugenol, kaempferol-3-β-D-glucopyranoside, p-coumaric acid and cinnamyl alcohol, were identified, with their retention times and contents being shown in Figure 2 and Table 1. As the peaks of quercetin-3-O-glucoside and quercetin-3-O-galactoside overlapped, they were quantified as the sum of two compounds to ensure methodological rigor and precision (Figure 2 and Table 1). But for CH, only five compounds, including benzoic acid, trans-cinnamic acid, cinnamaldehyde, eugenol and cinnamyl alcohol, were detected, with the contents being 4.2, 2.8, 1185.6, 13.7 and 4.3 μg/g, respectively (Table 1). This result conforms to a previously study by Wang et al. [3], showing that 15 compounds were identified with levels in the range of 0.3–17,985.2 μg/g in cinnamon leaf powder, as well as 5 compounds detected in CH, with levels being in the range of 1.62–1099.6 μg/g. By comparison, the total amount of 15 compounds in cinnamon leaf powder and 5 compounds in CH were higher than that reported by Wang et al. [3]. and Huang and Chen [2], which can be attributed to the discrepancy of cinnamon species growing in different locations of Taiwan, especially weather and environmental conditions.

### 3.2. Properties of Cinnamon Leaf Nanosuspension

**Figure 3:**
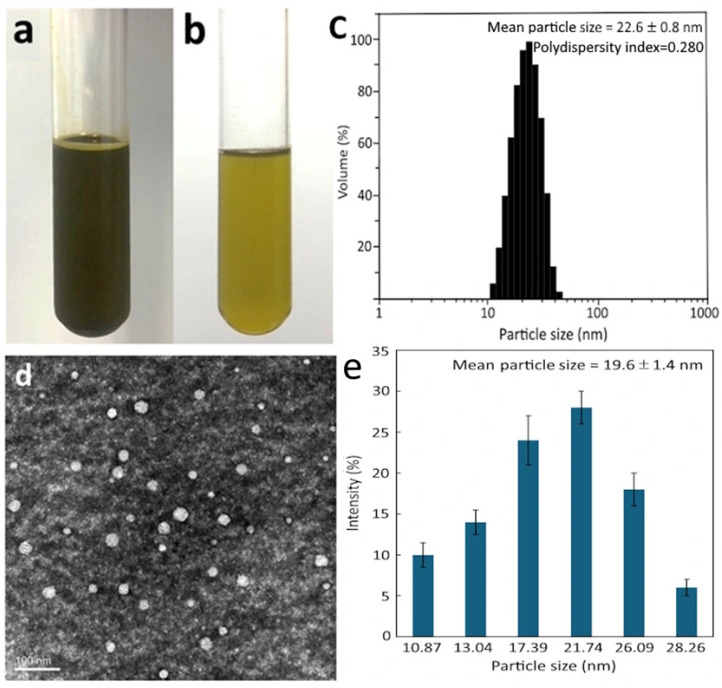
Appearance of CN (**a**) and diluted CN (**b**) as well as particle size distribution, PDI and zeta potential as determined by DLS method (**c**) and TEM image of CN with mean particle size at 19.6 nm (**d**,**e**). The methods for characterization analysis are provided in Section 2.4. CN, cinnamon leaf nanosuspension; PDI, polydispersity index; DLS, dynamic light scattering; TEM, transmission electron microscopy.

The CN was prepared successfully following the procedures described in the methods section, with the Figure 3a,b showing appearance in original and diluted forms, respectively. The mean particle size and polydispersity (PDI), as measured by a DLS instrument, was 22.6 nm and 0.280, respectively (Figure 3c), and the zeta potential, as measured by a zeta potential analyzer, was −49.5 mV. A low PDI of 0.280 implied a narrow distribution of nanoparticles in CN, while a large negative value of zeta potential (−49.5 mV) indicated a high stability of CN because of high electric charge repulsion force with the zeta potential at >30 mV or <−30 mV [3]. For TEM analysis, a mean particle size at 19.6 nm with a round shape was observed (Figure 3d,e). The difference in mean particle size measured by DLS and TEM can be attributed to the state of nanoparticles used for measurement, with the former carried out in a liquid suspension of nanoparticles for measuring the hydrodynamic size, consisting of a particle core along with the solvent layer attached to the particle as it moves in the liquid under the influence of Brownian motion, while the latter used dried nanoparticles for measuring the area of the sphere core [34]. Additionally, the encapsulation efficiency of CA was measured to be 91.6%, which is similar to that (90.8%) of CA in CN prepared by Wang et al. [3]. For storage stability, only a slight change in mean particle size (14.9–17.3 nm), PDI (0.187–0.263) and zeta potential (−40.8 to −49.5 mV) of CN was found over a 90-day storage period at 4 °C. Apparently, both surfactants Tween 80 and lecithin used for CN preparation should play a pivotal role in elevating the CN stability during storage. For stability under in vitro gastric and intestinal conditions, the CN showed an insignificant variation of particle size (23.2–24.7 nm) upon incubation with gastric fluid (Table 2). However, upon incubation with intestinal fluid, the particle size varied significantly between 29.1 and 33.2 nm. On the other hand, the zeta potential of CN changed significantly under both gastric and intestinal conditions, with the values, respectively, ranging from −30.8 to −34.2 mV and −38.1 to −40.8 mV at different incubation times (0–2 h) (Table 2). Moreover, compared to that before digestion (91.6%), the encapsulation efficiency dropped to 88.4% and 82.4%, respectively, after incubation with gastric and intestinal fluid, revealing a slight loss in encapsulated CA under the intestinal environment (Table 2). Nevertheless, these variations in the above physicochemical parameters are deemed minimal, ensuring the high stability of CN during storage and gastrointestinal conditions.

### 3.3. In Vitro Release Study

**Figure 4:**
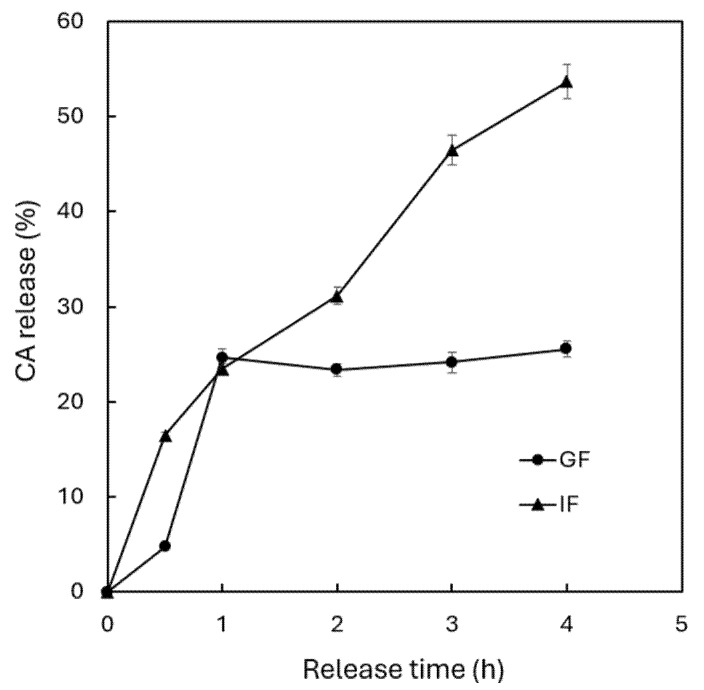
In vitro CA release from CN as a function of incubation time in simulated GF and IF for 4 h. Data plotted as mean ± standard deviation (*n* = 3). The methods for determination of in vitro release of CA from CN are provided in Section 2.5. CA, cinnamaldehyde; CN, cinnamon leaf nanosuspension; GF, gastric fluid; IF, intestinal fluid.

Figure 4 shows the in vitro release of CA from CN under simulated gastric condition for 2 h and intestinal condition for 4 h. Incubation of CN with simulated gastric fluid for 2 h showed a 24.6% release of CA in the initial 1 h and attained a plateau with a total CA release of 25.5% after 2 h of incubation. However, following the incubation of CN with the simulated intestinal fluid, a sustained release of CA was shown with the release rate continuously increasing, reaching a maximum of 53.7% after 4 h of incubation. This outcome implied that the release of CA from CN was significantly higher in intestine than in stomach. In other words, the CA is protected within CN even under the highly acidic gastric condition followed by a maximum release in the intestine for subsequent absorption via intestinal epithelial cells. A similar low release of phenolics from olive leaf extract-encapsulated nanoliposomes coated with chitosan was shown in the simulated gastric fluid (13%) when compared to that in the simulated intestinal fluid (60%) after 40 min of incubation for both at 37 °C [35]. In another study, an in vitro release of 8% and 95.4% of vitamin D3 was shown from nanoliposome encapsulated with vitamin D3 after incubating, respectively, in gastric and intestinal fluids for 2 h and 6 h at 37 °C [36]. This finding further demonstrates that through encapsulation of unstable compounds into a nanoliposome system, the stability of bioactive compounds in vivo could be greatly enhanced.

### 3.4. Type II Diabetes

**Figure 5:**
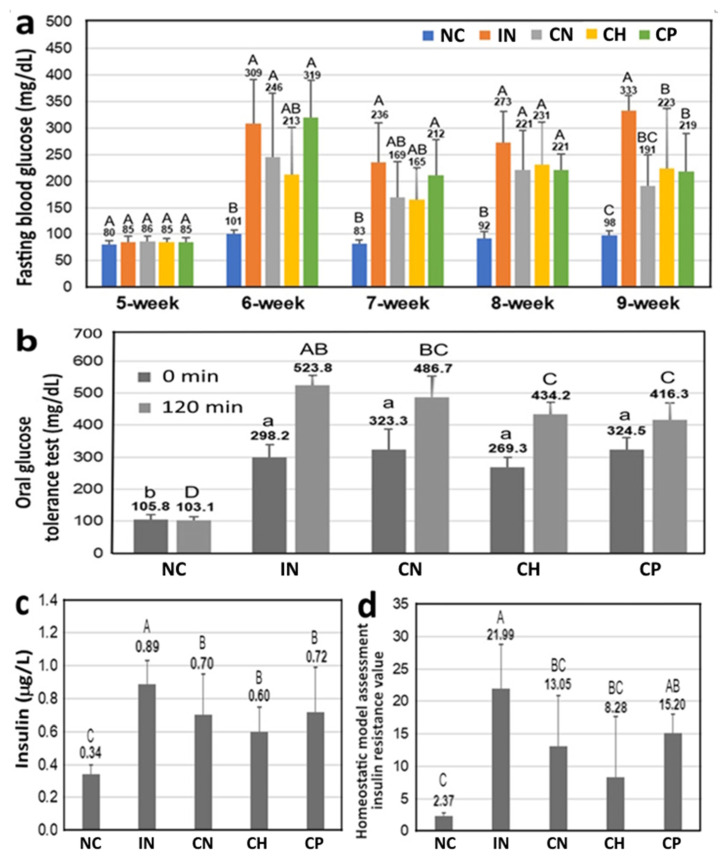
Effects of cinnamon leaf nanosuspension, hydrosol and powder in water on the levels of FBG (**a**), OGTT (**b**), insulin (**c**) and HOMA-IR (**d**) in T2D rats. Data presented as mean ± standard deviation (*n* = 6), and data bearing different capital letters (A–C) for FBG, insulin and HOMA-IR are significantly different among various treatments at *p* < 0.05, while data bearing different small letters (a–b) and different capital letters (A–D), respectively, at 0 min and 120 min for OGTT data are significantly different among various treatments at *p* < 0.05. NC, normal control group rats fed with deionized water by tube feeding each day for 9 weeks; IN, induction group rats fed with deionized water by tube feeding each day for 4 weeks, followed by induction of T2D once at the first day of the 5th week and continuing feeding until the 9th week; CN, cinnamon leaf nanosuspension group rats fed with nanosuspension containing CA at 60 mg/kg bw each day for 4 weeks, followed by induction of T2D at the first day of the 5th, 6th and 7th week for 3 times, and continuing feeding until the 9th week; CH, hydrosol group rats fed with hydrosol at 11.9 mg/kg bw each day for 4 weeks, followed by induction of T2D at the first day of the 5th, 6th and 7th week for 3 times, and continuing feeding until the 9th week; CP, powder group rats fed with cinnamon leaf powder in water at a dose of 0.5 g/kg bw each day for 4 weeks, followed by induction of T2D at the first day of the 5th, 6th and 7th week for 3 times, and continuing feeding until the 9th week; T2D, type II diabetes; bw, body weight; FBG, fasting blood glucose; OGTT, oral glucose tolerance test; HOMA-IR, homeostatic model assessment of insulin resistance.

Figure 5a shows the effect of CN, CH and CP on FBG levels in diabetic rats with high-fat diet and STZ induction, and data bearing different capital letters (A–C) within each week are significantly different among various treatments at *p* < 0.05. At the first day of the 5th week, the FBG levels after 12 h fasting were from 80–86 mg/dL for all the five groups, implying all the rats were healthy without T2D before induction. Following induction at the first day of the 5th week, the FBG levels were >200 mg/dL for IN, CN, CH and CP groups at the first day of the 6th week, revealing T2D was induced for these groups, with the leaf powder in water treatment showing the highest FBG level, followed by the IN, CN and CH treatments. To verify the successful induction of T2D, the groups CN, CH and CP were induced again. However, at the first day of the 7th week, the FBG levels of CN and CH groups declined to 169.0 and 164.8 mg/mL, respectively, while both IN and CP groups showed a FBG level > 200 mg/dL. Thus, groups CN, CH and CP were induced again with STZ at the first day of the 7th week, with the FBG levels being 231.2, 221.0 and 220.5 mg/dL at the first day of the 8th week, respectively, while that of IN group was 272.5 mg/dL. Compared with the IN group, which was only induced once to generate T2D, the other groups, including CN, CH and CP, were induced three times. This outcome clearly revealed that both CN and CH treatments were more effective in preventing T2D than CP treatment. Similarly, at the 9th week, with the exception of the NC group, the CN was the only treatment with the FBG level < 200 mg/dL. By comparison, based on the number of inductions, CN was the most efficient in preventing T2D, followed by CH and CP. Collectively, the cinnamon pretreatment exerts a preventive effect capable of delaying the onset of T2D.

The effect of CN, CH and CP on the blood glucose levels in diabetic rats with high-fat diet and STZ induction using an oral glucose tolerance test (OGTT) is shown in Figure 5b, and data bearing different small letters (a–b) and different capital letters (A–D), respectively, at 0 min and 120 min are significantly different among various treatments at *p* < 0.05. Only the IN group followed a time-dependent rise and reached a maximum OGTT value (523.8 mg/dL) at 120 min, while the other three groups, including CN, CH and CP, reached a peak OGTT value at 30 min, which equaled 520.6, 470.1 and 542.0 mg/dL, respectively, followed by a decrease to 486.7, 434.2 and 416.3 mg/dL, respectively, at 120 min. Compared with the IN group, the OGTT values were reduced by 7.1%, 17.1% and 20.5% for the CN, CH and CP treatments, respectively. It may be postulated that all the three treatments may elevate the β cell function of pancreatic islets for more insulin secretion to enhance glucose uptake in vivo.

The serum insulin levels in diabetic rats with high-fat diet and STZ induction are also shown in Figure 5c, and data bearing different capital letters (A–C) are significantly different among various treatments at *p* < 0.05. Compared with the IN group, the serum insulin levels for the CN, CH and CP treatments were diminished by 21.3%, 32.6% and 19.1%, respectively, with CH treatment showing the most pronounced effect. Similarly, for the HOMA-IR value, a decrease by 40.7%, 62.3% and 30.9% was shown for the CN, CH and CP treatments, respectively, compared with IN group (Figure 5d). Collectively, both CH and CN should be the most efficient in preventing T2D.

**Figure 6:**
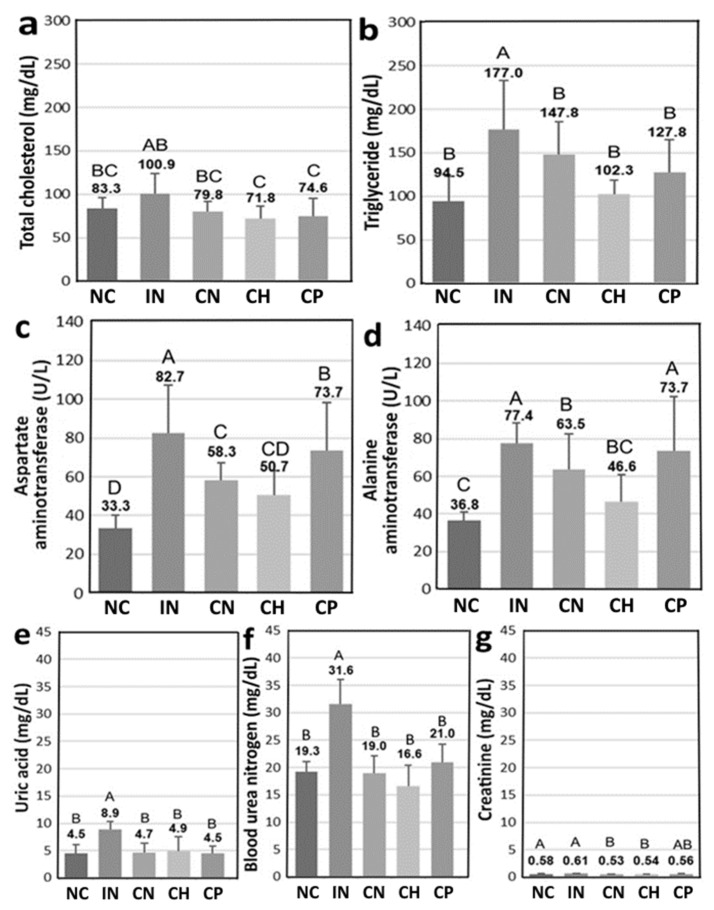
Effects of cinnamon leaf nanosuspension, hydrosol and powder in water on TC (**a**), TG (**b**), AST (**c**), ALT (**d**), URIC (**e**), BUN (**f**) and CREA (**g**) in the serum of T2D rats. Data presented as mean ± standard deviation (*n* = 6), and data bearing different capital letters (A–B) for TG, URIC, BUN and CREA, (A–C) for TC and ALT, and (A–D) for AST are significantly different among various treatments at *p* < 0.05. NC, normal control group rats fed with deionized water by tube feeding each day for 9 weeks; IN, induction group rats fed with deionized water by tube feeding each day for 4 weeks, followed by induction of T2D once at the first day of the 5th week and continuing feeding until the 9th week; CN, cinnamon nanosuspension group rats fed with nanosuspension containing CA at 60 mg/kg bw each day for 4 weeks, followed by induction of T2D at the first day of the 5th, 6th and 7th week for 3 times, and continuing feeding until the 9th week; CH, hydrosol group rats fed with hydrosol at 11.9 mg/kg bw each day for 4 weeks, followed by induction of T2D at the first day of the 5th, 6th and 7th week for 3 times, and continuing feeding until the 9th week; CP, powder group rats fed with cinnamon leaf powder in water at a dose of 0.5 g/kg bw each day for 4 weeks, followed by induction of T2D at the first day of the 5th, 6th and 7th week for 3 times, and continuing feeding until the 9th week; T2D, type II diabetes; bw, body weight; TC, total cholesterol; TG, triglyceride; AST, aspartate aminotransferase; ALT, alanine aminotransferase; URIC, uric acid; BUN, blood urea nitrogen; CREA, creatinine.

Figure 6 shows the biochemical parameters, including TC, TG, AST, ALT, URIC, BUN and CREA, in the serum of diabetic rats, and data bearing different capital letters (A–B) for TG, URIC, BUN and CREA, (A–C) for TC and ALT, and (A–D) for AST are significantly different among various treatments at *p* < 0.05. Compared with the IN group, the TC levels of the CN, CH and CP groups were, respectively, reduced by 20.8%, 28.8% and 26.0% (Figure 6a), as well as 16.5%, 42.2% and 27.8% for TG levels (Figure 6b). Likewise, compared with IN treatment, both AST and ALT levels in the serum of diabetic rats were decreased, respectively, by 29.5% and 18.0% for CN, 38.7% and 39.8% for CH, and 10.9% and 4.9% for CP (Figure 6c,d). A similar tendency was observed for URIC, BUN and CREA levels in the serum of diabetic rats, with a decline of 47.6%, 39.8% and 13.1% being shown, respectively, for CN treatment; 45.0%, 47.5% and 11.5% for CH treatment; and 49.3%, 33.5% and 8.2% for CP treatment, when compared with IN treatment (Figure 6e–g). All the biochemical parameter data further demonstrated that CN, CH and CP were effective in improving cardiovascular, liver and kidney functions without side effects.

**Figure 7:**
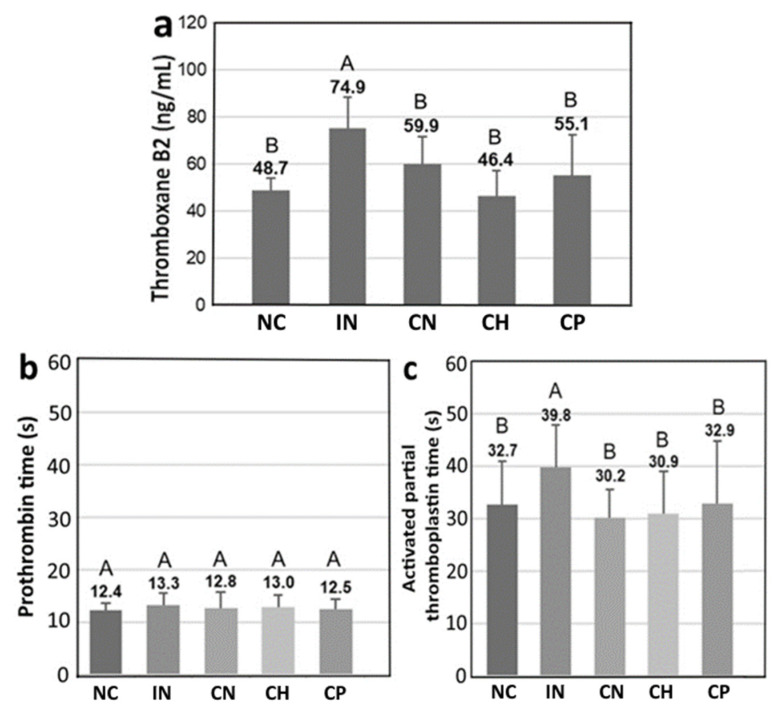
Effects of cinnamon leaf nanosuspension, hydrosol and powder in water on TXB2 (**a**), PT (**b**) and aPTT (**c**) in T2D rats. Data presented as mean ± standard deviation (*n* = 6), and data bearing different capital letters (A–B) for TXB2 and aPTT are significantly different among various treatments at *p* < 0.05, while PT data bearing the capital letter (A) for all the treatments represent insignificant difference among various treatments at *p* > 0.05. NC, normal control group rats fed with deionized water by tube feeding each day for 9 weeks; IN, induction group rats fed with deionized water by tube feeding each day for 4 weeks, followed by induction of T2D once at the first day of the 5th week and continuing feeding until the 9th week; CN, cinnamon nanosuspension group rats fed with nanosuspension containing CA at 60 mg/kg bw each day for 4 weeks, followed by induction of T2D at the first day of the 5th, 6th and 7th week for 3 times, and continuing feeding until the 9th week; CH, hydrosol group rats fed with hydrosol at 11.9 mg/kg bw each day for 4 weeks, followed by induction of T2D at the first day of the 5th, 6th and 7th week for 3 times, and continuing feeding until the 9th week; CP, powder group rats fed with cinnamon leaf powder in water at a dose of 0.5 g/kg bw each day for 4 weeks, followed by induction of T2D at the first day of the 5th, 6th and 7th week for 3 times, and continuing feeding until the 9th week; T2D, type II diabetes; bw, body weight; TXB2, thromboxane B2; PT, prothrombin time; aPTT, activated partial thromboplastin time (aPTT).

The values of prothrombin time (PT), activated partial thromboplastin time (aPTT) and thromboxane B2 (TXB2) are shown in Figure 7, and data bearing different capital letters (A–B) for TXB2 and aPTT are significantly different among various treatments at *p* < 0.05, while PT data bearing the capital letter (A) for all the treatments represent insignificant difference among various treatments at *p* > 0.05. Compared with the IN group, a reduction of TXB2 by 20.0%, 38.1% and 26.4% was found for CN, CH and CP, respectively, with CH showing the most distinct effect in decreasing TXB2 value (Figure 7a). A similar outcome was shown PT and aPTT, as evident by a decline of 3.5% and 24.1% for CN treatment, respectively, as well as 2.0% and 22.4% for CH treatment, and 6.2% and 17.3% for CP treatment (Figure 7b,c). This result indicates that all the three treatments could reduce blood coagulation time through an increase of blood flow and blood circulation.

### 3.5. Parkinson’s Disease

**Figure 8:**
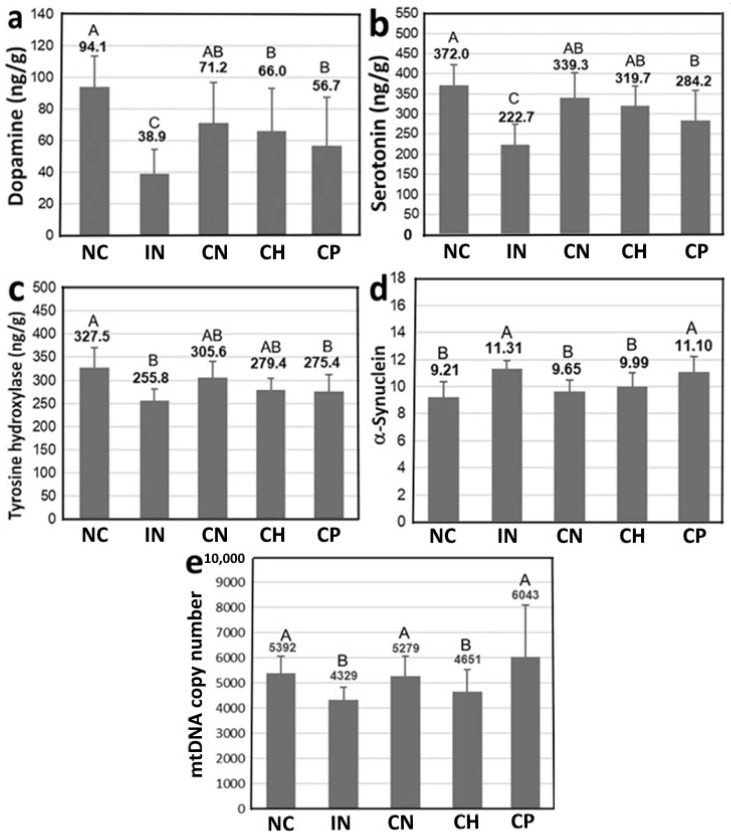
Effects of cinnamon leaf nanosuspension, hydrosol and powder in water on dopamine (**a**), serotonin (**b**), tyrosine hydroxylase (**c**), α-synuclein (**d**) and mtDNA copy number (**e**) in PD rat striatum. Data presented as mean ± standard deviation (*n* = 8), and data bearing different capital letters (A–C) for dopamine and serotonin as well as (A–B) for tyrosine hydroxylase, α-synuclein and mtDNA copy number are significantly different among various treatments at *p* < 0.05. NC, normal control group rats fed with deionized water by tube feeding each day for 4 weeks, followed by injecting sunflower oil (98%) and dimethyl sulfoxide (2%) subcutaneously at 1 mL/kg bw and then tube feeding with deionized water at 10 mL/kg bw for 5 weeks; IN, induction group rats fed with deionized water by tube feeding each day for 4 weeks, followed by injecting rotenone (dissolved in sunflower oil) subcutaneously at 2 mg/kg bw each day and then tube feeding with deionized water at 10 mL/kg bw each day for 5 weeks; CN, cinnamon leaf nanosuspension group rats were fed with nanosuspension by tube feeding 60 mg/kg bw each day for 4 weeks, followed by induction of PD, and continuing feeding with nanosuspension each dayf or 5 weeks; CH, hydrosol group rats fed with hydrosol by tube feeding each day at 11.9 mg/kg bw for 4 weeks, followed by induction of PD, and continuing feeding each day for 5 weeks; CP, powder in water group rats fed with powder in water by tube feeding at 0.5 g/kg bw each day for 4 weeks, followed by induction of PD and continuing feeding with powder in water each day for 5 weeks. PD, Parkinson’s disease; bw, body weight; mtDNA, mitochondria deoxyribonucleic acid.

Figure 8 shows the effects of CN, CH and CP on the levels of dopamine (a), serotonin (b), tyrosine hydroxylase (c), α-synuclein (d) and mtDNA copy number (e) in rats with PD induced with rotenone, and data bearing different capital letters (A–C) for dopamine and serotonin as well as (A–B) for tyrosine hydroxylase, α-synuclein and mtDNA copy number are significantly different among various treatments at *p* < 0.05. Compared with the IN group, a substantial rise in dopamine by 82.6%, 69.4% and 45.6% was shown for the CN, CH and CP treatments, respectively. As dopamine is a neurotransmitter and a vital index of PD, this result implied that CN was the most effective in improving PD in rats. Similarly, a large increase of serotonin, a pivotal neurotransmitter, by 52.3%, 43.5% and 27.6% was observed for CN, CH and CP treatments, respectively (Figure 8b). For tyrosine hydroxylase, an important enzyme capable of conversion of tyrosine into dopamine in the human brain, its levels were raised by 19.5%, 9.2% and 7.6%, respectively, following treatments with CN, CH and CP (Figure 8c). The results of both serotonin and tyrosine hydroxylase are obviously in accordance with the dopamine outcome. Another important soluble protein, α-synuclein, which can be expressed in the striatum of brain for subsequent accumulation to form Lewy bodies leading to PD, was found to decrease by 14.6%, 11.6% and 1.9%, respectively, for CN, CH and CP treatments, when compared with IN treatment (Figure 8d). But for mtDNA, a higher level by 21.9%, 7.4% and 39.5% was found for CN, CH and CP treatments, respectively (Figure 8e). This outcome implied that CP was the most effective in elevating the mtDNA level, essential for ATP production and cellular respiration, followed by CN and CH. However, no significant difference (*p* > 0.05) was observed between the CN and CP treatments. Apparently, based on these findings, CN showed the most prominent effect in preventing PD, followed by CH and CP. It may be inferred that the small particle size of CN (22.6 nm) can cross the blood–brain barrier more readily for activation of neuron cells, resulting in a preventive effect of PD [3,23].

**Figure 9:**
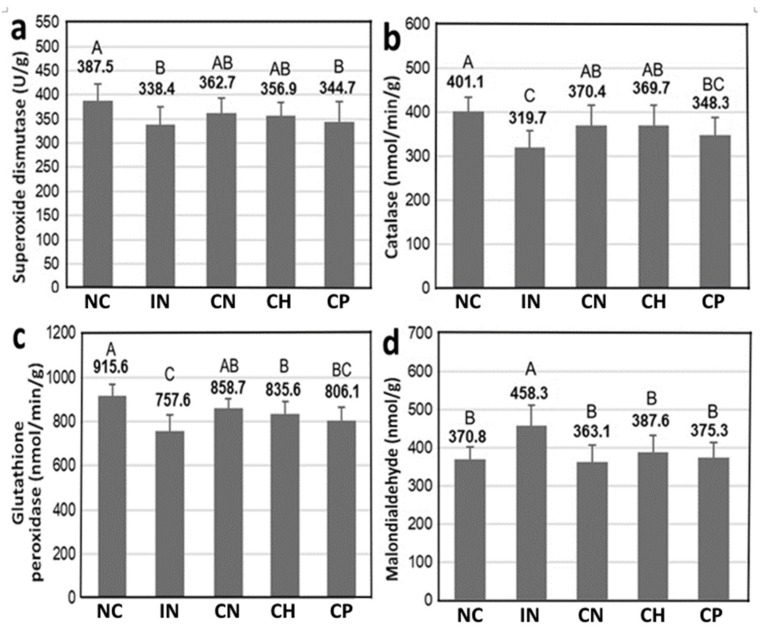
Effects of cinnamon leaf nanosuspension, hydrosol and powder in water on SOD (**a**), CAT (**b**), GSH-Px (**c**) and MDA (**d**) in PD rat midbrain. Data presented as mean ± standard deviation (*n* = 8), and data bearing different capital letters (A–B) for SOD and MDA as well as (A–C) for CAT and GSH-Px are significantly different among various treatments at *p* < 0.05. NC, normal control group rats fed with deionized water by tube feeding each day for 4 weeks, followed by injecting sunflower oil (98%) and dimethyl sulfoxide (2%) subcutaneously at 1 mL/kg bw and then tube feeding with deionized water at 10 mL/kg bw for 5 weeks; IN, induction group rats fed with deionized water by tube feeding each day for 4 weeks, followed by injecting rotenone (dissolved in sunflower oil) subcutaneously at 2 mg/kg bw each day and then tube feeding with deionized water at 10 mL/kg bw each day for 5 weeks; CN, cinnamon leaf nanosuspension group rats were fed with nanosuspension by tube feeding 60 mg/kg bw each day for 4 weeks, followed by induction of PD, and continuing feeding with nanosuspension each day or 5 weeks; CH, hydrosol group rats fed with hydrosol by tube feeding each day at 11.9 mg/kg bw for 4 weeks, followed by induction of PD, and continuing feeding each day for 5 weeks; CP, powder in water group rats fed with powder in water by tube feeding at 0.5 g/kg bw each day for 4 weeks, followed by induction of PD and continuing feeding with powder in water each day for 5 weeks. PD, Parkinson’s disease; bw, body weight; SOD, superoxide dismutase; CAT, catalase; GSH-Px, glutathione peroxidase; MDA, malondialdehyde.

The antioxidant enzyme activities, including SOD, CAT and GSH-Px, in the midbrain of rats with PD as affected by CN, CH and CP treatments are shown in Figure 9, and data bearing different capital letters (A–B) for SOD and MDA as well as (A–C) for CAT and GSH-Px are significantly different among various treatments at *p* < 0.05. Compared with the IN treatment, the SOD activity rose by 7.2%, 5.5% and 1.9% for the CN, CH and CP treatments, respectively (Figure 9a). A similar trend was found for CAT, as evident by an increment of 15.9%, 15.6% and 8.9%, respectively, for the CN, CH and CP treatments (Figure 9b). Likewise, the GSH-Px activity was raised by 13.3%, 10.3% and 6.4%, respectively, following treatment with CN, CH and CP (Figure 9c). But for MDA, a major lipid degradation product generated from lipid hydroperoxides during lipid oxidation, it was shown to decline by 20.8%, 15.4% and 18.1% for CN, CH and CP treatments, respectively, when compared with IN treatment (Figure 9d). All these results further revealed that CN was the most efficient in elevating antioxidant activity in the rat midbrain, followed by CH and CP. As reactive oxygen species produced during lipid oxidation has been corroborated to be an imperative factor in causing PD, the antioxidant activity data shown in this study further demonstrated the potential preventive effect of CN and CH prepared from cinnamon leaves.

**Figure 10:**
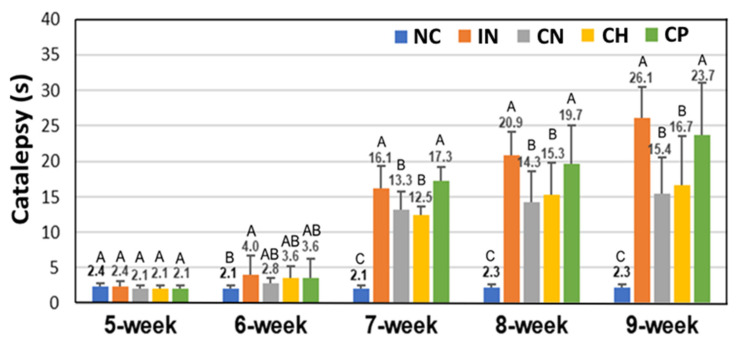
Effects of cinnamon leaf nanosuspension, hydrosol and powder in water on catalepsy test data in PD rats. Data presented as mean ± standard deviation (*n* = 8), and data bearing different capital letters (A–C) within each week are significantly different among various treatments at *p* < 0.05. NC, normal control group rats fed with deionized water by tube feeding each day for 4 weeks, followed by injecting sunflower oil (98%) and dimethyl sulfoxide (2%) subcutaneously at 1 mL/kg bw and then tube feeding with deionized water at 10 mL/kg bw for 5 weeks; IN, induction group rats fed with deionized water by tube feeding each day for 4 weeks, followed by injecting rotenone (dissolved in sunflower oil) subcutaneously at 2 mg/kg bw each day and then tube feeding with deionized water at 10 mL/kg bw each day for 5 weeks; CN, cinnamon nanosuspension group rats were fed with nanosuspension by tube feeding 60 mg/kg bw each day for 4 weeks, followed by induction of PD, and continuing feeding with nanosuspension each day or 5 weeks; CH, hydrosol group rats fed with hydrosol by tube feeding each day at 11.9 mg/kg bw for 4 weeks, followed by induction of PD, and continuing feeding each day for 5 weeks; CP, powder in water group rats fed with powder in water by tube feeding at 0.5 g/kg bw each day for 4 weeks, followed by induction of PD and continuing feeding with powder in water each day for 5 weeks. PD, Parkinson’s disease; bw, body weight.

Figure 10 shows the effect of CN, CH and CP on catalepsy test data (expressed as sec) in rats with PD, and data bearing different capital letters (A–C) within each week are significantly different among various treatments at *p* < 0.05. Compared with the IN group, a decreasing tendency was observed following treatments with CN, CH and CP over a 5-week period, with a maximum drop by 41.0%, 36.1% and 9.5% at the 9th week, respectively. This finding further demonstrated that the CN was the most effective in preventing PD through a substantial reduction in catalepsy time, followed by CH and CP. This outcome further supported the finding of a recent study dealing with improving PD in rats by CN and byproducts prepared from cinnamon leaves by Wang et al. [3], showing that a high-dose CN at 60 mg/kg bw was efficient in treating rats with PD. Our findings in this study further strengthen the evidence of cinnamon leaf-derived products in preventing PD.

### 3.6. Comparative Discussion on T2D and PD Disease Biomarkers as Well as Their Association

#### 3.6.1. Type II Diabetes

Many animal and human studies dealing with the anti-diabetic effects from cinnamon and its products have been published. In a recent study, Huang and Chen [2] prepared CH, extract and nanoemulsion from cinnamon leaves and compared their protection effects on STZ-induced T2D in rats, with the high-dose CN (60 mg/kg bw) showing the most pronounced effect in reducing FBG, OGTT, serum insulin and insulin resistance index levels, followed by low-dose nanoemulsion (20 mg/kg bw), high-dose extract (60 mg/kg bw), low-dose extract (20 mg/kg bw) and leaf powder in hydrosol (0.5 g/10 mL). As CA is the dominant bioactive compound in cinnamon leaves, this anti-diabetic effect can be attributed to CA and the presence of some other phytochemicals such as eugenol and cinnamyl alcohol through a synergistic effect. Additionally, it has been well documented that oxidative stress, caused by the overproduction of ROS and an inadequate amount of antioxidants in vivo, can lead to diabetes through the production of proinflammatory cytokines such as tumor necrosis factor-alpha (TNF-α), interleukin-1β (IL-1β) and interleukin-6 (IL-6) for subsequent impairment of cell membrane and reaction with DNA, lipid and protein [37,38]. Also, CA was shown to raise the CAT and GSH activities in STZ-induced diabetic rats while mitigating the levels of TNF-α, NO and MDA [39]. Thus, the amelioration of oxidative stress through the intake of a combination of CA and other phytochemicals can be another approach in preventing T2D. Among the various treatments, the CN was the most effective in preventing T2D in rats, which should be due to the presence of a small mean particle size (22.6 nm) of CN for penetration into pancreatic β cells more readily for the optimal control of insulin secretion. Interestingly, in another study designed to explore the effects of cinnamic acid and CA on T2D rats, cinnamic acid, but not CA, decreased FBG levels and improved glucose tolerance [40]. This discrepancy may be attributed to the low dose of CA (10 mg/kg bw), length of feeding period, and induction mode. Additionally, a large conversion of CA to cinnamic acid in rats may account for this negative effect as well [3].

TXB2, a vital index of platelet hyperaggregability, has been found to be closely associated with oxidative stress in diabetic patients, caused by the elevation of lipid peroxidation products such as MDA [41]. Specifically, oxidative stress can lead to a rise in phospholipase-A2 activity for the subsequent release of arachidonic acid and the induction of platelet activation and production of thromboxane-A2 (TXA2), responsible for platelet aggregation, clot formation and smooth muscle contraction, with TXB2 being a stable metabolite of TXA2 [41,42]. As a significant reduction of MDA in rat brains following the administration of CN, CH and CP was observed in our study, this finding further demonstrated that the intake of cinnamon-derived products may reduce the risk of platelet aggregability and thrombotic disease. In addition to thrombosis, the measurement of both blood glucose and TXB2 levels has been suggested to predict the progress of patients with ischemic stroke [43]. Thus, it can be inferred that the cinnamon products prepared in our study may provide a protective effect against ischemic stroke.

Blood circulation, functioning as the transportation system for nutrients, oxygen and waste removal in our body, is crucial in maintaining human health, especially for heart and brain health, and wound healing. One of the main issues in patients with T2D is poor blood circulation, as high blood glucose level can result in the formation of fatty deposits between the skin and muscle, which, in turn, decreases blood flow, caused by the hardening and narrowness of blood vessels, a phenomenon called atherosclerosis [44]. This syndrome can also be observed in diabetic patients with peripheral artery disease. Moreover, normal blood flow is pivotal for efficient hemostasis, a process of stopping bleeding, as it ensures the delivery of coagulation factors such as proteins to the injury site for clotting, while slow blood flow can prolong blood coagulation time, elevating the risk of thrombosis and T2D. Additionally, slow blood circulation can result in tissue hypoxia, while fast blood circulation can lead to the elevation of cardiovascular burden. Thus, maintaining a normal blood flow rate through the intake of cinnamon-derived products can be another approach to minimize complications associated with T2D while increasing the chances of bioactive compounds in CN to enter the brain.

Prothrombin time (PT), a crucial indicator of blood’s clotting ability affecting blood circulation directly, can be used to measure the activity of the extrinsic and common coagulation pathway, while the activated partial thromboplastin time (aPTT) can be used to assess the integrity of the intrinsic and common pathways [45]. As prothrombin is a protein produced by the liver, playing a pivotal role in the blood clotting process, an optimal time length of PT (11.4–16.6 s) and aPTT (21–35 s) for rats should be controlled. In our study, both PT (12.5–13.0 s) and aPTT (30.2–32.9 s) for rats were properly controlled for CN, CH and CP treatments. Significantly, a prolonged PT may reveal vitamin K deficiency, liver problems or certain bleeding disorders, while a shortened PT may indicate high risk of blood clots and thrombosis. Furthermore, a high blood flow rate may result in a prolonged PT caused by difficulty in promoting aggregation of clotting factor and platelet, which is pivotal for hemostasis to occur. Similarly, a shortened aPTT may elevate the thrombosis risk, while a prolonged aPTT may indicate a deficiency in certain clotting factors [46,47]. As shortened aPTT may arise from an accumulation of circulating activated coagulation factors in plasma caused by enhanced coagulation activation in vivo, aPTT can be used to assess the risk of thromboembolic complications in patients with diabetes mellitus [48,49]. Most importantly, this finding demonstrates that CN, CH and CP treatment may be effective in the prevention of bleeding complications associated with diabetic patients as high blood sugar can damage blood vessels, leading to retinal capillary hemorrhage. Moreover, an overproduction of thrombus can consume a large quantity of clotting factors and platelet, resulting in hemorrhage.

For human studies, it was shown that cinnamon polyphenols improved insulin sensitivity through a reduction of FBG (18–29%), total cholesterol (12–26%), triacylglycerol (23–30%) and low-density lipoprotein (LDL)-cholesterol (7–27%) in subjects with T2D after daily consumption of cinnamon at 1–6 g daily for 40 days [49]. In addition, an aqueous extract (high in type A polyphenols) also demonstrated improvements in FBG, glucose tolerance and insulin sensitivity in women with insulin resistance associated with polycystic ovary syndrome [49]. In another clinical trial designed to evaluate the effect of cinnamon bark powder supplementation with 500 mg capsules twice daily for 3 months on anthropometric, glycemic and lipid outcomes of patients with T2D, all anthropometric parameters, including body mass index (BMI), body fat and visceral fat, glycemic indices including fasting plasma glucose, 2 h postprandial glucose, glycated hemoglobin, fasting insulin and insulin resistance, as well as lipids, including total cholesterol, LDL-cholesterol and high-density lipoprotein (HDL)-cholesterol, were significantly improved [50]. More recently, Zelicha et al. [51] explored the effects of daily cinnamon spice supplementation at 4 g/day for 4 weeks used for seasoning on glucose concentrations in adults with obesity and prediabetes, a significant reduction of 24 h glucose concentrations, mean net-area-under-the-curve for glucose and triglyceride, as well as an increase of glucose-dependent-insulinotropic-polypeptide concentration during oral glucose tolerance testing plus cinnamon testing were observed when compared with placebo group. This finding further demonstrates that the intake of cinnamon may contribute to better glucose control when added to the diet in people who have obesity-related prediabetes. However, only 18 subjects participated in this clinical trial, which should be inadequate. Nevertheless, the outcome of this study provides a basis for a possible large scale of clinical trials in the future dealing with the therapeutic treatment of patients with T2D by cinnamon-derived products.

#### 3.6.2. Parkinson’s Disease

Tyrosine hydroxylase, mainly present in the adrenal medulla, central nervous system and peripheral sympathetic neurons, is a regulatory enzyme in the synthesis of catecholamine for the subsequent production of L-3,4-dihydroxyphenylalanine (L-DOPA), a precursor of dopamine, in dopaminergic neurons in the presence of oxygen, ferrous ion and tetrahydrobiopterin through tyrosine hydroxylation [52,53]. More specifically, tyrosine hydroxylase can be activated by phosphorylation-dependent binding to 14-3-3 proteins, which were reported to be associated with chronic neurodegenerative diseases such as PD and Alzheimer’s disease [54]. Thus, the elevation of tyrosine hydroxylase activity is a vital approach in preventing and treating PD.

α-synuclein, a protein composed of 140 amino acids, can aggregate to form oligomers and produce amyloid fibrils known as Lewy bodies, a key step in the development of PD [55]. In a study dealing with inhibition of α-synuclein oligomeric and fibrillar assembly in PD using a Drosophila fly model expressing mutant A53T α-synuclein in the nervous system, an aqueous cinnamon extract precipitation was shown to possess a significant curative effect on the behavioral symptoms of flies and on α-synuclein aggregation in their brain [55]. In our study, we also observed a substantial reduction of α-synuclein in the striatum of rats when administered with CN or CH, revealing that both products were effective in preventing the occurrence of PD.

In this study, we measured the levels of dopamine, serotonin, tyrosine hydroxylase, α-synuclein and mtDNA in the striatum of rat brains, as well as SOD, CAT, GSH-Px and MDA in rat midbrains. It is well known that dopamine plays a crucial role in the striatum, a brain region involved in learning, reward and movement. Specifically, dopamine can help regulate movement and influence motor function through the nigrostriatal pathway, a major dopaminergic pathway by projecting from the substantia nigra to the striatum, and thereby, the progressive loss of dopamine can lead to PD as the presence of dopamine is essential for neurotransmission in the corpus striatum [3,53]. Moreover, dopamine can be released through communication of neurons in basal ganglia with nigral neurons, with this interaction being imperative for the fine-tuning of movements [53].

The major symptoms of PD can be divided into motor and non-motor, with the former including tremor, rigidity, bradykinesia and postural instability, while the latter includes dementia, depression, fatigue and sleep disturbances [56]. However, over the last decade, several studies have suggested that serotonergic dysfunction can be associated with the development of motor and non-motor symptoms and complications. Specifically, serotonin cell bodies are located in the raphe nuclei of the brainstem and can be divided into the rostral group and the caudal group, with the former projected to the forebrain innervating amygdala, hypothalamus, cingulum, medial cerebral cortex, basal ganglia and part of hippocampus, and the latter projected to spinal cord and caudal brainstem [57,58,59]. Thus, the impairment of serotonergic neurotransmission may also be responsible for the non-motor and motor symptoms associated with PD. Additionally, previously published reports have suggested that caudal brainstem serotonergic neurons can be affected before dopaminergic neurons in the midbrain, implying the possibility of both nervous systems to be affected simultaneously [59]. The elevation of serotonin levels in the striatum of rat brains following the administration of CN and CH in our study further demonstrated a possible preventive effect on PD following the intake of both products. Like dopamine, serotonin, a happy hormone synthesized in the brainstem for attenuation of depression, can be converted to melatonin (hormone of darkness) in the pineal gland of the brain for the regulation of sleep–wake cycles.

As mentioned above, dysfunctional mitochondria have been suggested to play a key role in neuronal death, as neuronal energy is provided by mitochondria through oxidative metabolism. Moreover, as mtDNA is essential for ATP production by the respiratory chain, mtDNA alterations in the brain can lead to neurodegeneration-associated diseases such as PD and Alzheimer’s disease. Specifically, genetic studies on familial PD have discovered monogenic forms with disease-causing mutations in several genes leading to mitochondrial dysfunction, including PARK2, PARK6, PARK7 and PARK8 [60]. This finding provides direct evidence demonstrating the association between mitochondrial damage and the occurrence of PD. Consequently, the measurement of mtDNA levels in the striatum of rats can be a crucial index in assessing the prevention or improvement of PD.

In addition to α-synuclein deposits, some other mechanisms involved in the pathogenesis of PD include neuroinflammation, oxidative stress, ferroptosis, mitochondrial dysfunction, gut dysbiosis and autophagy regulation, in which oxidative stress should play an imperative role in the progression of PD [61,62]. Oxidative stress is a phenomenon arising from an imbalance between the production and accumulation of reactive oxygen species (ROS) in cells and tissues and the capability of an antioxidant to detoxify ROS. The excessive production of free radicals can then damage cell membrane, lipid, protein and DNA, leading to oxidative injury. Among the various antioxidants in the human brain, glutathione is an important one, with its level being shown to be reduced in the substantia nigra of patients with PD for the subsequent impairment of mitochondria function, probably caused by production of hydrogen peroxide and ROS during dopamine metabolism [63,64]. Moreover, NADPH oxidase and mitochondria were reported to be the most important ROS generators, both playing a pivotal role in inducing oxidative stress, resulting in neurotoxicity [62]. Thus, the alleviation of oxidative stress can be another important approach for PD prevention and treatment.

Likewise, many animal and clinical trial reports dealing with anti-PD have been documented. In an MPTP (1-methyl-4-phenyl-1, 2, 3, 6-tetrahydropyridine)-induced mouse model of PD, cinnamon powder was shown effective in upregulating Parkin and DJ-1, both of which can protect dopaminergic neurons from damage, thereby achieving an improved effect in PD [65]. Interestingly, sodium benzoate, a frequently used food preservative, was produced in the brain and blood of mice following the oral feeding of cinnamon powder for the subsequent suppression of proinflammatory cytokines such as IL-1β and iNOS [65,66]. Sodium benzoate, generated from cinnamic acid through β-oxidation in the liver, is a component of Ucephan, a drug approved by the USFDA for the treatment for hepatic metabolic defects associated with hyperammonemia such as urea cycle disorder in children [67,68]. As CA, the dominant compound in cinnamon, can be converted to cinnamic acid by oxidation in vivo, both CA and sodium benzoate should play a key role in protecting against dopaminergic cell death, motor deficits and striatal neurotransmitter dysregulation through various mechanisms, including the retardation of proinflammatory cytokines, upregulation of DJ-1 and Parkin, elevation of antioxidant activity and glial-derived neurotrophic factor, as well as the regulation of autophagy and the TLR/NFκB pathway [69,70]. More elaborately, in the striatum of normal C57/BL6 and aged A53T-α-syn transgenic mice, the oral feeding of sodium benzoate was shown to enhance the expression of tyrosine hydroxylase, a rate-limiting enzyme in the dopamine biosynthesis pathway, accompanied by an upregulation of striatal dopamine and an improvement of locomotor activity [53].

#### 3.6.3. Association Between Type II Diabetes and Parkinson’s Disease

The association between T2D and PD has been well documented. However, a contradictory result still exists regarding the role of T2D on the risk and progression of PD. Sandyk [71] was the first to report that patients with both PD and T2D suffered more serious motor symptoms and a reduced response to drug treatment. Since then, many clinical studies have been conducted to verify that patients with T2D had a higher risk of developing PD [72,73]. If true, the current drugs used for treatment of T2D could be used for cure of PD. For instance, both anti-T2D drugs exenatide (a glucagon-like peptide-1 agonist) and dipeptidyl peptidase were shown to be effective in improving PD symptoms, but a conflicting outcome was reported with metformin, a popular drug for the treatment of T2D [73]. This finding further substantiates the therapeutic complexity of PD when coexisting with T2D. It may be postulated that anti-T2D drugs mainly act on peripheral tissues such as liver, muscle and adipose cells to elevate sensitivity toward insulin for the subsequent transportation of insulin to these cells and decrease of blood sugar level. Furthermore, anti-diabetic drugs may fail to cross BBB maintaining normal brain cell function. Thus, the possible alleviation of PD symptoms through the intake of anti-T2D drugs may result from an indirect route with only a small portion of drugs capable of entering the brain. Nevertheless, there is abundant evidence showing a positive relationship between T2D and PD. For example, a decline in dopamine transporter level was found in diabetic mice [26]. Furthermore, mitochondrial impairment occurred in an insulin knockout or insulin-resistant mice model, leading to a rise in phosphorylated α-synuclein, a major component of Lewy bodies [27,74,75].

The BBB, a physiological barrier between blood and the brain, controls the movement of substances from blood and prevents nearly all large molecules and 98% of small molecules (drugs) from entering the brain [76]. However, lipophilic molecules with MW less than 400 Da were shown to cross the BBB readily through lipid-mediated diffusion [77]. Structurally, the BBB is composed of endothelial cells, astrocytes, pericytes and a basement membrane, with the tight junctions between adjacent endothelial cells providing ultrahigh transendothelial resistance [76]. Specifically, tight junctions can prevent the passive diffusion of hydrophilic substances through paracellular transport, the passage of solutes from blood into the brain parenchyma by passing through the intercellular space between cells [77]. Instead, hydrophilic compounds need to cross the BBB by mechanisms of transcellular transport involving active transport by specific carriers [77]. Thus, to remedy the problem associated with crossing the BBB by hydrophilic or lipophilic compounds, the development of a nanosystem such as nanoliposome or nanoemulsion with an optimal size is pivotal [3,23,78]. The possible pathways of nanoparticles in crossing the BBB have been shown to include the lipophilic transcellular pathway, hydrophilic paracellular pathway, functionalized nanoparticle-carrier mediated pathway, ionized-nanoparticle adsorptive trancytosis and ligand-nanoparticle receptor-mediated transcytosis [79]. As the CN prepared in our study is composed of soybean oil, Tween 80, lecithin, water and cinnamon leaf extract with a mean particle size of 19.6 nm as measured by TEM and 22.6 nm by DLS, a combination of various pathways may be involved in crossing the BBB. More elaborately, both lipophilic lecithin and hydrophilic Tween 80 can cross the BBB through binding with apolipoprotein E (ApoE) in blood for the subsequent conjugation with LDL on the brain cell membrane. Similarly, lecithin, a precursor of a vital neurotransmitter acetylcholine, was shown to bind with ApoE for conjugation with the brain cell membrane receptor by the subsequent uptake by endothelial cells via transcytosis for delivering bioactive compounds to the brain [3,80]. But for the essential fatty acids present in soybean oil, linolenic acid (5–9%) and linoleic acid (49–53%), both can cross the BBB by passive diffusion through transport using transmembrane protein and transcytosis for maintaining the integrity of brain cell membrane and nerve functions [23,81]. As for the cinnamon leaf extract, containing the major bioactive compounds such as CA, cinnamic acid, cinnamyl alcohol and eugenol, they can cross the BBB readily due to lipophilic nature with MW < 400 Da as mentioned above. Most importantly, a mean particle size of 22.6 nm of CN should be able to cross the BBB more efficiently while avoiding the opsonization effect of the reticuloendothelial system as reported by Ohta et al. [82], showing that a nanoparticle size at 15 nm was more effective than at 3 nm, probably caused by the opsonization effect of the latter.

In addition, hyperglycemia was shown to result in an increment of advanced glycation end product in vivo, formed through the reaction of lipid, protein or nucleic acid with sugar in a non-enzymatic process, leading to chronic disease, particularly diabetes, as a result of the aging process [28]. More importantly, the advanced glycation end products were present in Lewy bodies via aggregation with phosphorylated α-synuclein [28]. As no significant association between diabetes duration and the risk of PD was observed, a feasible explanation is that a high risk of PD may occur for clinical trial subjects with poor glycemic control and complications for T2D [73]. Namely, the contradictory outcomes may result from clinical trial subjects with various degrees of severity of T2D.

Among the various biomarkers used for PD evaluation, the lipid oxidation products, such as MDA, and prooxidants, such as iron, were shown to rise substantially in the brains with PD, with the main accumulation sites being Lewy bodies in PD [23]. As both MDA and prooxidants are responsible for oxidative stress and play a vital role in PD pathogenesis, the elevation of antioxidant enzyme activities, including SOD, CAT and GSH-Px, following treatment with CN, CH and CP, are expected to improve PD as observed in this study (Figure 9). Obviously, the antioxidant CA in CN, CH and CP can elevate the production and function of antioxidant enzymes, including SOD, CAT and GSH-Px, by upregulating their gene expressions through stress-response pathways (Nuclear factor-2 erythroid related factor-2, Nrf2) [83]. These upregulated antioxidant enzymes efficiently neutralize ROS responsible for oxidative stress into hydrogen peroxide, which is further broken down into harmless water and oxygen, thereby protecting cells from oxidative damage [83]. In addition to reducing the oxidative stress, oxygen depletion, a vital pathological feature in both T2D and PD patients, can be alleviated through oxygen generation during oxidative stress reduction by CA. Oxygen depletion in PD pathology results in disrupting mitochondrial function, increasing α-synuclein aggregation and impairing dopaminergic neurons [84], while that in T2D pathology contributes to the development of disease, impairing essential cellular functions through vascular damage, impaired red blood cells, thrombus formation/leukostasis, systemic hypoxia and insulin resistance [85]. The capabilities of oxygen-supplied nanomaterials in breaking the hypoxic barrier have been explored in a recent review article by Xiang et al. [86].

It is interesting to note that diabetic peripheral neuropathy (DPN), the most common type of neuropathy, affecting about 50% of diabetic patients, can impair the nerves outside the brain and spinal cord, particularly in the hands and legs, with the symptoms being motor neuropathy, sensory neuropathy or both, leading to atrophy, cramping, twitching, muscle weakness and other movement-associated problems [47]. Interestingly, the motor symptoms observed in diabetic patients can also occur in PD patients. It was reported that DPN is more prevalent in people with PD than in the general population, with the motor symptoms being worsened and the risk of falls being elevated [29]. Specifically, DPN prevalence in patients with PD varies from 4.8% to 55%, compared with 9% in the general population [29]. However, it remains unclear if peripheral neuropathy can lead to increased balance deficits and impaired mobility in PD patients. Nevertheless, this finding further revealed that diabetic patients possess a high risk of developing DPN, ultimately leading to PD. Therefore, it is imperative to develop a cinnamon-derived product providing a protective effect on both T2D and PD.

Regarding the signal pathways involved in PD and T2D progression, insulin has been demonstrated to control peripheral activities in the brain by the autonomic nervous system and the hypothalamic-pituitary axis through action on the hippocampus and prefrontal cortex to mitigate depression symptoms and enhance cognitive function [87], which can be associated with PD. Furthermore, insulin can be released into blood by pancreatic β cells, with insulin receptors (IR) being expressed in the basal ganglia and substantia nigra, both of which are critical areas in the brain for patients with PD [87]. In other words, patients with PD possessed greater insulin resistance and less insulin receptor mRNA in their substantia nigra pars compacta [88,89]. It may be postulated that the reduction of insulin in PD patients can be linked to the suppression of the IR-IRS-1-PI3K/AKT pathway in the hippocampus region of the brain, which, in turn, elevates the levels of phosphorylated IRS-1 at serine residues 636 and 616 [87]. Additionally, the IRS-1 phosphorylation on serine residues could prevent insulin/IGF-1 from binding to the IR and the subsequent activation of downstream effectors as it is a crucial element of functional insulin signaling. The additional downstream effectors of PI3K-AKT include nuclear factor kappa-light-chain-enhancer of activated B cells (NFkB), forkhead box protein O1 (FOXO1), mammalian target of rapamycin (mTOR) and glycogen synthase kinase 3β (GSK3β) [72]. Some other pathways, such as activation of the IR-Shc-MAP kinase (MAPK) pathway, involved in promoting the genetic expression of proteins for cell growth and synapse plasticity, are also pivotal, as insulin has been shown to act on this pathway for the regulation of translation, transcription and post-translational modification of proteins, as well as to affect learning and memory [90,91]. Another important pathway involved is the direct activation of NMDA glutamate receptors to increase the opening of calcium channels at synapses and promote NMDA-mediated neurotransmission for a subsequent rise in the recruitment of γ-aminobutyric acid (GABA) receptors to postsynaptic sites for the elevation of GABA transmission, thereby regulating synaptic inhibition for neuronal function involved in learning and memory [92,93]. Thus, insulin resistance can be connected to a faster PD progression through the stimulation of α-synuclein aggregation. Nevertheless, a reactive metabolite generated during hyperglycemia in diabetes mellitus, methylglyoxal, was shown to cause the oligomerization of α-synuclein, which is more toxic than larger aggregations of α-synuclein [55]. In addition to insulin resistance, some other parameters, such as advanced glycation end products, microglial activation and inflammation, are also imperative in the progression of PD and T2D.

Furthermore, in addition to the PI3K/AKT pathway and MAPK pathway, some other pathways, including the AMPK, UPR, WNT, TGFβ, HIFs, EGFs, bile acids, Hippo and Ca^2+^-related signaling pathways, are involved in insulin resistance and/or β cell dysfunction [94]. As these signaling pathways enable the regulation of biological processes dealing with insulin production and controlling of glucose metabolism, it is feasible to develop a product for T2D prevention by targeting a specific pathway. For instance, a DPP3 inhibitor in the plasma was found to increase GLP-1 content by inhibiting GLP-1 degradation by DPP4, while the agonists of GLP-IR could promote insulin secretion in pancreatic β cells through different signaling pathways [94]. Similarly, for PD, many pathways, including PI3K-AKT/mTOR, MAPK, HO-1, PLCr, NLRP3/caspase-1/gasdermin D and the BDNF/TrkB/cyclic AMP response element binding protein, are involved [23]. More importantly, CA was demonstrated to improve T2D and PD through the activation of the PI3K/AKT and MAPK/ERK pathways [95], and thereby, the prevention of T2D and PD through intake of a cinnamon-derived product is feasible. Nevertheless, the association between prevention of T2D/PD and intake of CN/CH/CP by controlling various signal pathways needs to be further explored.

In this study, the preventive effects of CN, CH and CP have been demonstrated in T2D and PD rat models. Furthermore, a comparable effect was shown between post-administered treatment (reported in our previous studies) [2,3] and pre-administered treatment (in this study) in improving T2D and PD in rat models. For instance, in a previous study dealing with improving STZ-induced T2D in rats by nanoemulsion, hydrosol and extract prepared from cinnamon leaves, Huang and Chen [2] reported that the levels of FBG, insulin, OGTT and HOMA-IR were reduced by 21.4–54.2%, 8.9–47.8%, 25.0–55.7% and 23.6–76.0%, respectively, for nanoemulsion, hydrosol and extract treatments, with the high-dose nanoemulsion (60 mg/kg bw) being the most effective. Similarly, in another study dealing with the effects of nanoemulsion and byproducts (hydrosol and extract) on improving PD in rats, Wang et al. [3] demonstrated that the high-dose nanoemulsion (60 mg/kg bw) was the most efficient as evident by a reduction of α-synuclein by 16.0% and catalepsy time by 61.5%, as well as an elevation of dopamine by 49.4% and tyrosine hydroxylase by 25.6%. By comparison, in the present study the levels of FBG, insulin, OGTT and HOMA-IR were reduced by 33.0–42.6%, 19.1–32.6%, 7.1–20.5% and 30.9–40.7% for high-dose CN (60 mg/kg bw). But for PD, a substantial increase in dopamine by 45.6–82.6% and tyrosine hydroxylase by 9.2–19.5%, accompanied by a decline in α-synuclein by 1.9–14.6% and catalepsy time by 9.5–41.0%, were shown for CN, hydrosol and powder in water. Collectively, both pre-administered results in this study and post-administered results from previous studies verified a comparable effect in improving TD2 and PD using a rat model. But it should be pointed out that multiple injections of STZ/NA for treatment groups of CN, CH and CP versus a single STZ/NA injection for the induction group should have demonstrated a better prevention effect in the present study.

However, there are several limitations that need further investigation in future studies. Although the possible biochemical pathways involved in the preventive action have been discussed above, the specific biochemical pathways responsible for the observed effects remain to be explored as the preventive action of CN, CH and CP on T2D and PD progression may modulate key signaling pathways related to insulin sensitivity, oxidative stress and inflammation, with Nrf2 and NFkB being the prominent shared pathways [96,97,98]. Additionally, T2D and PD share dysregulated processes, including mitochondrial dysfunction, dysregulation of glucose metabolism and protein aggregation [97]. Both methylglyoxal resulting from hyperglycemia and α-synuclein glycation resulting from PD pathogenesis are found in elevated levels in T2D patients, suggesting a link in molecular mechanism between T2D and PD [97]. Such links also necessitate the exploration of glucose variability commonly observed among T2D patients on PD development and progression. Furthermore, several key protein expressions, including adiponectin, C-reactive protein, interleukin-6 and ApoE involved in T2D progression [99], as well as neurofilament light chain (NfL), Glial fibrillar acidic protein (GFAP), DJ-1, glucocerebrosidase (GCase), Leucine-Rich Repeat Kinase 2 (LRRK2), tau and parkin in PD progression [100], are required to be measured in T2D and PD rats to deepen our understanding on the preventive action of CN, CH and CP. While murine (mouse and rat) models are essential for early biomedical research, they suffer significant limitations, necessitating human clinical trials. These limitations originate primarily from fundamental differences in genetics, physiology and disease manifestation between rodents and humans. More specifically, although animal and human share many genes, their expression and regulation vary significantly, resulting in differences in the metabolic rates, immune system responses and drug metabolism pathways. Additionally, animal models may fail to predict some serious human side effects and do not usually address complex cognitive, environmental and lifestyle factors in diverse human population. Thus, by conducting human clinical trials, the existing challenges in the treatment, including safety and efficiency, and alleviating responses in humans, can be overcome while verifying the findings observed in this study.

## 4. Conclusions

In conclusion, in this study, we demonstrated the preventive effects of cinnamon leaf nanosuspension, hydrosol and powder in water on T2D and PD in rats, with nanosuspension possibly being the most prominent in preventing PD. But for the prevention of T2D, nanosuspension, hydrosol and powder in water were comparable, while no side effect was observed, and blood circulation was optimized for these treatments. This finding demonstrates a great potential of cinnamon leaf-derived products in preventing the occurrence of chronic diseases such as T2D and PD. However, we need to point out here that repeated STZ/NA injection to induce T2D itself may introduce additional physiological stress or metabolic disturbances, which could confound the interpretation of results. Thus, some more clinical trials need to be conducted to verify the preventive effect observed in this study.

## Figures and Tables

**Table 1:** Identification and quantitation data of bioactive compounds in cinnamon leaf powder and hydrosol by UPLC-MS/MS.

Peak No.	Compound ^a^	Retention Time (min)	MS/MS (*m*/*z*)		Content (µg/g) ^c^	
		Sample	Precursor Ion	Production	Powder	Hydrosol ^d^
1	Quercetin	8.45	301	151	21.6 ± 1.8	ND ^e^
2	Coumarin	5.68	147	91	3.1 ± 0.2	ND
3 ^b^	Quercetin-3-*O*-galactoside Quercetin-3-*O*-glucoside	7.40	463	300	5.7 ± 0.4	ND
4	Rutin	7.32	609	300	3.9 ± 0.2	ND
5	Caffeic acid	4.29	179	134	3.3 ± 0.1	ND
6	Benzoic acid	6.69	121	77	61.5 ± 0.8	4.2 ± 0.9
7	5-*O*-Caffeoylquinic acid	2.15	353	191	0.7 ± 0.2	ND
8	*trans*-Cinnamic acid	9.20	147	103	435.7 ± 8.3	2.8 ± 0.1
9	Cinnamaldehyde	8.45	132	55	19,546.8 ± 271.5	1185.6 ± 70.5
10	Kaempferol	8.19	287	153	5.3 ± 0.6	ND
11	Eugenol	10.84	165	137	225.6 ± 3.7	13.7 ± 0.3
12	Kaempferol 3-β-D-glucopyranoside	8.47	447	284	23.8 ± 1.5	ND
13	*p*-Coumaric acid	5.67	164	90	5.4 ± 0.7	ND
14	Cinnamyl alcohol	8.29	117	115	96.8 ± 5.7	4.3 ± 0.3

^a^ Peaks were positively identified by comparison of retention time and mass spectra of unknown peaks with standards. ^b^ Both peaks were overlapped with the content being 5.7 μg/g for quercetin-3-*O*-galactoside plus quercetin-3-*O*-glucoside. ^c^ Data are presented as mean ± standard deviation of triplicate analyses. ^d^ Hydrosol was analyzed directly by UPLC-MS/MS without solvent extraction. ^e^ ND, not detected.

**Table 2:** Mean particle size, zeta potential of CN and encapsulation efficiency of CA as affected by incubation time in simulated gastric and intestinal fluids.

Incubation Time (h)	Particle Size (nm) ^a^	Zeta Potential (mV) ^a^	Encapsulation Efficiency (%) ^a^
In GF			
0	23.2 ± 0.5 ^D^	−33.2 ± 0.9 ^DE^	– ^b^
0.5	23.6 ± 0.9 ^D^	−34.2 ± 0.9 ^D^	–
1	24.1 ± 1.2 ^D^	−32.8 ± 0.3 ^E^	–
1.5	23.2 ± 0.8 ^D^	−32.5 ± 0.9 ^E^	–
2	24.7 ± 1.2 ^D^	−30.8 ± 0.3 ^F^	88.4 ± 0.3 ^A^
In IF			
0	29.1 ± 1.2 ^C^	−39.6 ± 0.3 ^AB^	–
0.5	31.2 ± 0.2 ^B^	−40.8 ± 1.2 ^A^	–
1	29.7 ± 1.4 ^BC^	−38.1 ± 0.2 ^C^	–
1.5	30.9 ± 0.6 ^BC^	−39.3 ± 1.0 ^BC^	–
2	33.2 ± 1.5 ^A^	−38.3 ± 0.6 ^BC^	82.4 ± 0.3 ^B^

^a^ Data shown are mean ± standard deviation (*n* = 3), and data bearing different letters (A–F) in the same column are significantly different at *p* < 0.05. ^b^ Dash sign (–) represents data not detected. CN, cinnamon leaf nanosuspension; CA, cinnamaldehyde; GF, simulated gastric fluid; IF, simulated intestinal fluid.

## Data Availability

The original contributions presented in this study are included in the article. Further inquiries can be directed to the corresponding author.

## List of Abbreviations

ALT	alanine aminotransferase
AMPK	adenosine monophosphate activated kinase
ANOVA	analysis of variance
ApoE	apolipoprotein E
aPTT	activated partial thromboplastin time
AST	aspartate aminotransferase
BBB	Blood–brain barrier
BDNF/TrkB	brain-derived neurotrophic factor (BDNF)/tropomyosin receptor kinase B
BMI	body mass index
BUN	blood urea nitrogen
bw	body weight
CA	cinnamaldehyde
CAT	catalase
CH	cinnamon leaf hydrosol
CN	cinnamon leaf nanosuspension
CP	cinnamon leaf powder
CREA	creatinine
DJ-1	Parkinson-associated protein deglycase
DLS	dynamic light scattering
DPN	diabetic peripheral neuropathy
DPP4	dipeptidyl peptidase-4
EGFs	epidermal growth factors
ERK	extracellular signal regulated kinase pathway
FBG	fasting blood glucose
FOXO1	forkhead box protein O1
GABA	γ-aminobutyric acid
GCase	glucocerebrosidase
GFAP	glial fibrillar acidic protein
GLP-1	glucogen like peptide 1
GSH-Px	glutathione peroxidase
GSK3β	glycogen synthase kinase 3 beta
HDL	high density lipoprotein
HIFs	hypoxia-inducible factors
HO-1	heme oxygenase-1
HOMA-IR	homeostatic model assessment of insulin resistance
IACUC	Institutional Animal Care and Use Committee
IGF-1	insulin-like growth factor 1
IL-1β	interleukin-1 beta
IL-6	interleukin-6
IN	induction
iNOS	inducible nitric oxide synthase
IR	insulin resistance
IR-IRS-1-PI3K/AKT	insulin resistance-insulin receptor substrate 1-phosphoinositide-3 kinase/protein kinase B
IR-Shc-MAP	insulin receptor-Src homology and collagen protein-mitogen-activated protein (MAP) kinase
LDL	low density lipoprotein
L-DOPA	L-3,4-dihydroxyphenylalanine
LRRK2	Leucine-Rich Repeat Kinase 2
MAPK	mitogen-activated protein kinase
MDA	malondialdehyde
MPTP	1-methyl-4-phenyl-1, 2, 3, 6-tetrahydropyridine
MRM	multiple reaction monitoring
mtDNA	mitochondrial deoxyribonucleic acid
mTOR	mammalian target of rapamycin
MW	molecular weight
NA	nicotinamide
NADPH	nicotinamide adenine dinucleotide phosphate
NC	normal control
ND	not detected
NfL	neurofilament light chain
NFκB	nuclear factor kappa-light-chain-enhancer of activated B cells
NLRP3	nod like receptor family pyrin domain containing 3
NMDA	N-methyl-D-aspartate
NO	nitric oxide
Nrf2	Nuclear factor-2 erythroid related factor-2
OGTT	oral glucose tolerance test
PARK2, PARK6, PARK7, PARK8	Parkinson proteins
PD	Parkinson’s disease
PDI	polydispersity index
PLCr	platelet large cell ratio
PT	prothrombin time
ROS	reactive oxygen species
SAS	statistical analysis system
SOD	superoxide dismutase
STZ	streptozotocin
T2D	type II diabetes
TC	total cholesterol
TEM	transmission electron microscopy
TFDA	Taiwan food and drug administration
TG	triglycerides
TGFβ	transforming growth factor-beta
TLR/NFκB	toll-like receptor/nuclear factor kappa-light-chain-enhancer of activated B cell
TNF-α	tumor necrosis factor-alpha
TXB2	thromboxane B2
UPLC-MS/MS	ultra-performance liquid chromatography-tandem mass spectrometry
UPR	unfolded protein response
URIC	uric acid
USFDA	United States Food and Drug Administration
WNT	wingless-related integrated site
